# Parkin inhibits proliferation and migration of bladder cancer via ubiquitinating Catalase

**DOI:** 10.1038/s42003-024-05935-x

**Published:** 2024-02-29

**Authors:** Renjie Zhang, Wenyu Jiang, Gang Wang, Yi Zhang, Wei Liu, Mingxing Li, Jingtian Yu, Xin Yan, Fenfang Zhou, Wenzhi Du, Kaiyu Qian, Yu Xiao, Tongzu Liu, Lingao Ju, Xinghuan Wang

**Affiliations:** 1 Department of Urology, Laboratory of Precision Medicine, Zhongnan Hospital of Wuhan University, Wuhan, China; 2 Department of Biological Repositories, Human Genetic Resources Preservation Center of Hubei Province, Hubei Key Laboratory of Urological Diseases, Zhongnan Hospital of Wuhan University, Wuhan, China; 3 Euler Technology, ZGC Life Sciences Park, Beijing, China; 4 Center for Quantitative Biology, School of Life Sciences, Peking University, Beijing, China; 5 Department of Urology, Peking University Aerospace Center Hospital, Beijing, China; 6 Department of Radiology, Zhongnan Hospital of Wuhan University, Wuhan, China; 7 Medical Research Institute, Frontier Science Center for Immunology and Metabolism, Taikang Center for Life and Medical Sciences, Wuhan University, Wuhan, China

**Keywords:** Bladder cancer, Ubiquitylation, Tumour-suppressor proteins

## Abstract

*PRKN* is a key gene involved in mitophagy in Parkinson’s disease. However, recent studies have demonstrated that it also plays a role in the development and metastasis of several types of cancers, both in a mitophagy-dependent and mitophagy-independent manner. Despite this, the potential effects and underlying mechanisms of Parkin on bladder cancer (BLCA) remain unknown. Therefore, in this study, we investigated the expression of Parkin in various BLCA cohorts derived from human. Here we show that *PRKN* expression was low and that *PRKN* acts as a tumor suppressor by inhibiting the proliferation and migration of BLCA cells in a mitophagy-independent manner. We further identified Catalase as a binding partner and substrate of Parkin, which is an important antioxidant enzyme that regulates intracellular ROS levels during cancer progression. Our data showed that knockdown of *CAT* led to increased intracellular ROS levels, which suppressed cell proliferation and migration. Conversely, upregulation of Catalase decreased intracellular ROS levels, promoting cell growth and migration. Importantly, we found that Parkin upregulation partially restored these effects. Moreover, we discovered that USP30, a known Parkin substrate, could deubiquitinate and stabilize Catalase. Overall, our study reveals a novel function of Parkin and identifies a potential therapeutic target in BLCA.

## Abstract (web summary)

A study on bladder cancer indicates that Parkin influences bladder cancer progression and metastasis by regulating cellular ROS levels through ubiquitin-mediated proteasomal degradation of Catalase, rather than its autophagic function.

## Introduction

As the global population ages, cancer is predicted to become the leading cause of death worldwide by 2030^1,2^. Bladder cancer (BLCA) is projected to be the fourth most common cancer in men in the United States, with death from BLCA ranking eighth by 2023^3^. BLCA is classified into non-muscle-invasive bladder cancer (NMIBC) and muscle-invasive bladder cancer (MIBC) based on clinical characteristics. NMIBC has a better prognosis but is more prone to relapse, while MIBC diagnosis is often accompanied by distant metastases, leading to a poor prognosis^4^. The incidence of BLCA is closely related to risk factors such as smoking and exposure to benzidine. Increasing evidence suggests that the imbalance between oxidants and antioxidants may play a pivotal role in the development of BLCA^5^. Therefore, gaining a comprehensive understanding of the underlying biological mechanisms of BLCA progression and metastasis is crucial in discovering new therapeutic approaches.

The ubiquitin protease system (UPS) is a critical epigenetic modification involved in the entire process of tumor development and progression^6^. Parkin, encoded by the *PRKN* gene, is an E3 ubiquitin ligase initially identified in autosomal recessive inherited juvenile Parkinson’s disease^7^. Subsequent research revealed that phosphorylation of Parkin by phosphatase and tensin homolog (PTEN)-induced kinase 1 (PINK-1) has a neuroprotective function by removing damaged mitochondria through mitophagy^8^. In recent years, increasing studies and reviews have emphasized a potentially more significant role of Parkin in cancer^9–11^, one that depends on its well-known function in mitophagy^12,13^, while the other relies on its crucial role as an E3 ubiquitin ligase, acting as an antitumor factor by directly or indirectly affecting genes in some critical pathways^10,14–16^. Notably, Parkin is downregulated in multiple cancers^17,18^. Although one study reported the involvement of Parkin in mitophagy in BLCA^19^, the effects of Parkin on the biological behavior of BLCA cells were not explored.

Oxidative stress has been recognized as a significant factor in the occurrence and development of tumors^20,21^. Reactive oxygen species (ROS), which include hydroxyl radicals (·OH), superoxide anions radicals (·O_2_^-^), and hydrogen peroxide (H_2_O_2_), are considered a double-edged sword. Physiologically, ROS play a crucial role in organisms. Excessive ROS can damage proteins and DNA through oxidative damage, causing many diseases, including cancer. ROS can cause cancer cells to die in high concentrations^22,23^. Catalase, a relatively ancient redox protein, is mainly involved in regulating ROS (mainly H_2_O_2_), which is important for maintaining intracellular redox levels^24,25^. However, the role of Catalase in cancer is still controversial, and its expression varies in multiple cancers. For instance, Catalase has been observed to have higher expression in chronic lymphocytic leukemia^26^, melanoma^27^, gastric carcinoma^28^, and glioma^29^. In contrast, the expression level or activity of Catalase is decreased in pancreatic cancer^30^, prostate carcinoma^31^, acute myeloid leukemia^32^, colorectal cancer^33^, and non-melanoma skin cancer^27^. In BLCA, lower expression and activity of Catalase have been reported^34,35^. However, recent studies found that a significant increase in Catalase activity was observed in BLCA patients compared to controls^36,37^. Clearly, the role of Catalase in BLCA requires further investigation.

Ubiquitin-specific protease 30 (USP30) is a protein located in the mitochondrial outer membrane and peroxisomes and is characterized by a unique transmembrane domain^38^. As a ubiquitin-specific deubiquitinase, USP30 cleaves the Lys6-ubiquitin chain^39^ and Lys11-linked polysomes^40^. Studies have shown that USP30 regulates peroxisomal autophagy independently of PINK1 and Parkin^41^. Moreover, USP30 has been found to stabilize DRP1, promote liver tumor growth^42^, and regulate tumor metabolism^43^. However, USP30 can also antagonize Parkin in the autophagy pathway^44,45^. Therefore, investigation of the role of USP30 in BLCA could be intriguing.

Clinical and epidemiological evidence strongly suggests that *PRKN* is an important tumor suppressor gene in several types of cancer, and low expression levels of *PRKN* are associated with poor prognosis. In this study, we have demonstrated that *PRKN* functions as a tumor suppressor gene in BLCA by reducing Catalase through the ubiquitination of Catalase and reducing USP30, which in turn stabilizes Catalase. This regulation of ROS inhibits the proliferation and migration of BLCA cells, which implies that targeting the Parkin-USP30-Catalase pathway could be a potential molecular therapy for BLCA. Our findings pave the way for further studies investigating the role of Parkin in BLCA and the development of novel treatment strategies for BLCA patients.

## Results

### PRKN has low expression and poor prognosis in BLCA

**Fig. 1:**
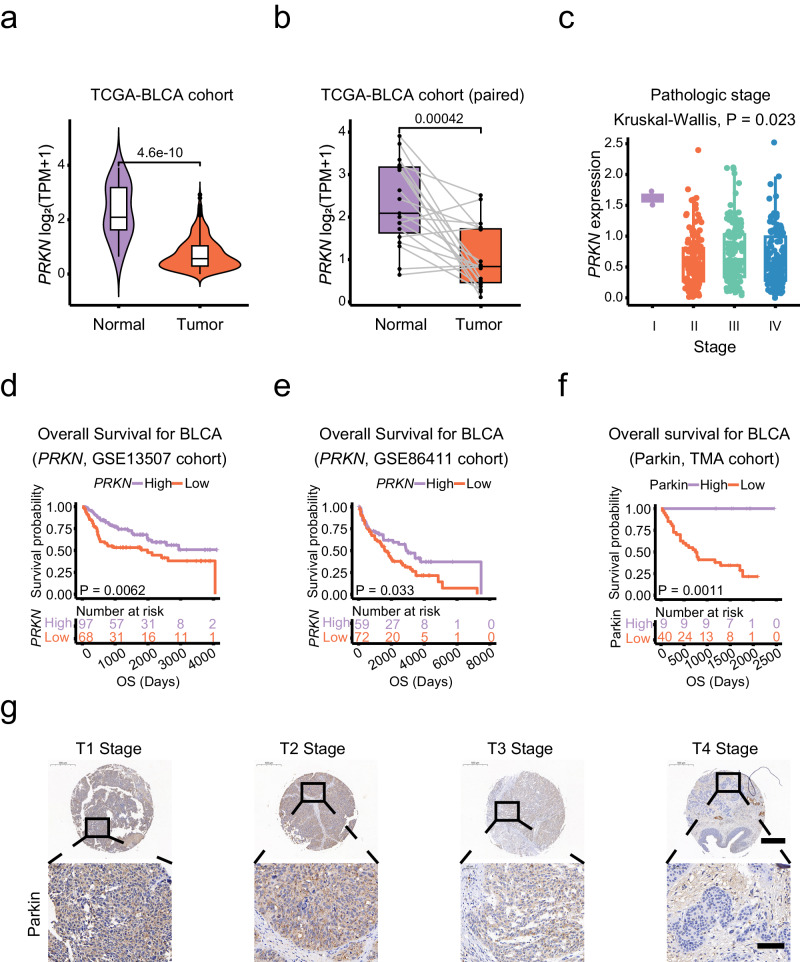
Expression level of the *PRKN* gene in BLCA. **a** *PRKN* expression levels in BLCA (*n* = 404) and control (*n* = 19) samples determined by using the Wilcoxon test, *p* = 4.6e-10. Data were obtained from the TCGA database. **b** Comparison of *PRKN* expression levels in BLCA (*n* = 19) versus paired samples (*n* = 19) by using the Wilcoxon test, *p* = 0.00042. Data were obtained from the TCGA database. **c** The relationship between *PRKN* expression and BLCA stage was determined by using the Kruskal-Wallis test, *p* = 0.023, and the data were obtained from the TCGA database. **d** Survival analysis was used to explore the relationship between *PRKN* expression level and BLCA prognosis (*p* = 0.0062). The data were obtained from the GSE13507 dataset (*n* = 165). **e** Survival analysis was used to explore the relationship between *PRKN* expression level and BLCA prognosis (*p* = 0.033). The data were obtained from the GSE86411 dataset (*n* = 131). **f** Survival analysis was used to explore the relationship between Parkin expression level and BLCA prognosis (*p* = 0.0011). The data were obtained from 63 human BLCA samples. **g** IHC showed that Parkin were detected in different T stages from the samples of human BLCA in TMA, scale bar: 0.5 mm, 100 μm (enlarged).

We first analyzed the expression of *PRKN* in BLCA using data from TCGA (https://tcga-data.nci.nih.gov/tcga/). The data indicate that *PRKN* expression is reduced by half in BLCA tissues compared to adjacent normal tissues (Fig. 1a). This finding was consistent in 19 paired samples, including cancer and adjacent carcinoma samples (Fig. 1b), and we found that *PRKN* expression differed according to pathological stage (Fig. 1c). Furthermore, *PRKN* was also found to be expressed at low levels in carcinoma tissues in three different BLCA datasets (Supplementary Fig. 1a). Survival analysis showed that low *PRKN* expression was significantly associated with a poorer prognosis (Fig. 1d, e and Supplementary Fig. 1b, c). To confirm this, we analyzed immunohistochemical (IHC) samples from 63 BLCA patients and observed low expression of Parkin in cancer (Supplementary Fig. 1d). Similarly, patients in the low expression of Parkin group had a worse outcome in TMA cohort (Fig. 1f). We observed the expression of Parkin in both paracancerous tissue and tumor tissue from the same patient through IHC staining (Supplementary Fig. 1e). We found that low Parkin expression was correlated with pathological grade, muscle invasion and T stage (Supplementary Table 1). Furthermore, to further verify the diagnostic value of Parkin expression, univariate Cox analysis showed that risk factors for poor prognosis in BLCA patients included pathological grade, muscle invasion, N stage and low Parkin expression (Supplementary Table 2). Meanwhile, multivariate Cox analysis showed that risk factors for poor prognosis in BLCA patients included pathological grade, muscle invasion, and low Parkin expression (Supplementary Table 3). In addition, we discovered that the expression of Parkin in T4 stage BLCA samples and NMIBC samples decreased (Fig. 1g and Supplementary Fig. 1f, g). Finally, mRNA level analysis of 9 pairs of BLCA and adjacent normal tissues from Zhongnan Hospital revealed lower levels of Parkin in the tumor samples (Supplementary Fig. 1h). These results suggest that low Parkin expression is closely associated with poor prognosis in BLCA patients.

### PRKN plays a role as a tumor suppressor in BLCA

**Fig. 2:**
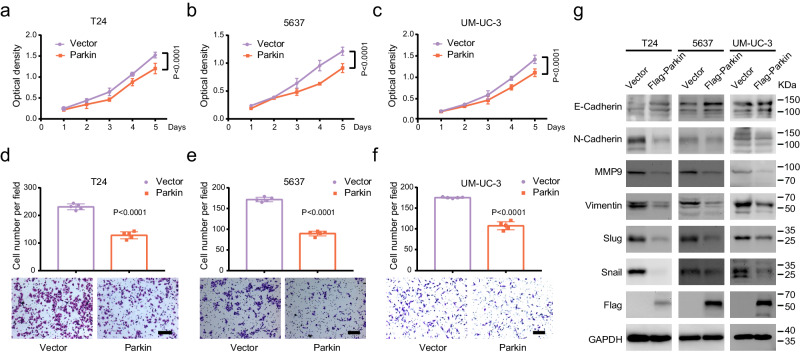
Overexpression of Parkin suppresses BLCA cell proliferation and migration. The MTT assay showed the proliferation of T24 (**a**), 5637 (**b**), and UM-UC-3 (**c**) cells following Parkin overexpression (*n* = 8, two-way ANOVA). Transwell assays displayed the migration of T24 (**d**), 5637 (**e**), and UM-UC-3 (**f**) cells after overexpression of Parkin (scale bar: 200 μm, *n* = 5, unpaired two-tailed Student’s t-test). **g** Western blot analysis showed the change in EMT-related proteins in Parkin upregulation in T24, 5637, and UM-UC-3 cells. Data are the mean ± SD. Exact *p* values are shown. The n number represents n biologically independent experiments in each group.

We initially assessed the transcriptional and protein expression levels of Parkin in immortalized uroepithelial cell line SV-HUC-1 and six commonly used BLCA cell lines using qRT-PCR and Western blot assay (Supplementary Fig. 1i). To investigate the potential function of Parkin in BLCA, we transiently transfected empty vector and Parkin into human BLCA cell lines (T24, 5637, and UM-UC-3 cells). Overexpression of Parkin was verified to be efficient (Supplementary Fig. 2a). We then evaluated the proliferation and migration ability of the cells using MTT and clonogenic assays, which demonstrated that Parkin overexpression significantly inhibited proliferation in T24, 5637, and UM-UC-3 cells (Fig. 2a–c and Supplementary Fig. 2b–d). Transwell and wound healing assays showed that Parkin overexpression markedly reduced the migration capacity of T24, 5637, and UM-UC-3 cells (Fig. 2d–f and Supplementary Fig. 2e, f). Western blot analysis revealed that Parkin overexpression significantly reduced the expression of mesenchymal-derived proteins, such as N-Cadherin, MMP9, Slug, Snail, and Vimentin during EMT while increasing the expression of epithelial-derived E-Cadherin (Fig. 2g). These results suggest that *PRKN* plays a role as a tumor suppressor by inhibiting proliferation and migration in BLCA.

### Parkin upregulation was not associated with autophagy but increased intracellular ROS

**Fig. 3:**
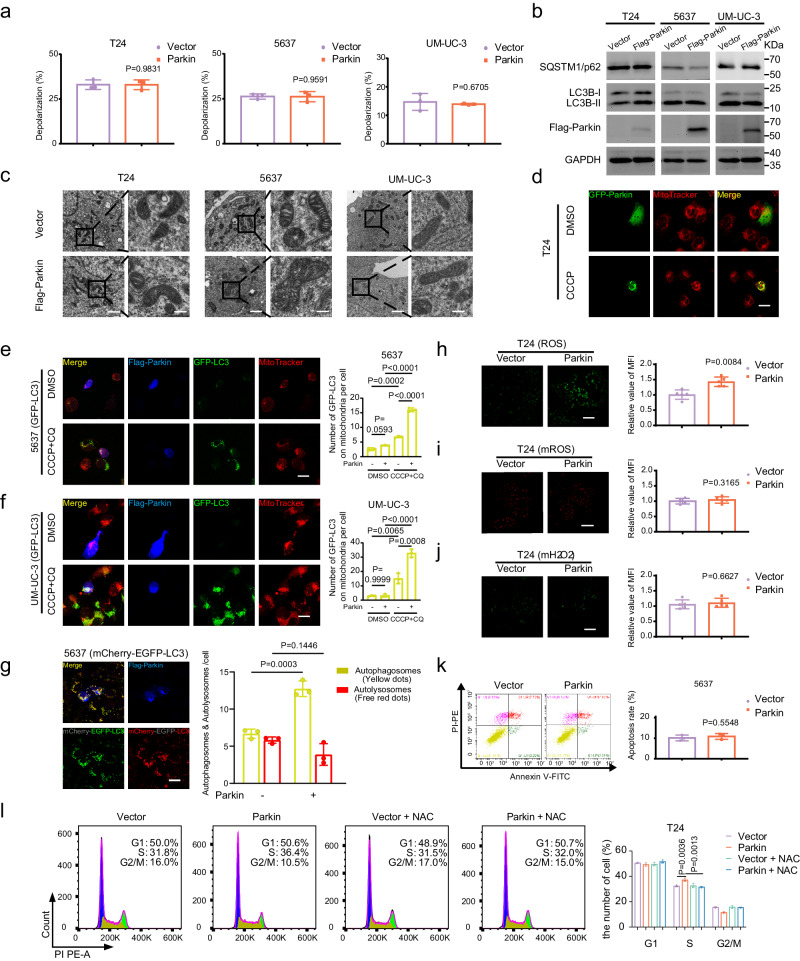
Overexpression of Parkin increased intracellular ROS levels in an autophagy-independent manner. **a** The depolarization levels of the mitochondrial membrane potential in T24, 5637, and UM-UC-3 cells were analyzed by flow cytometry (*n* = 3, unpaired two-tailed Student’s t-test). **b** Western blot showing the expression of autophagy-related proteins (SQSTM1/p62 and LC3B-I/II) in Parkin upregulation in T24, 5637, and UM-UC-3 cells. **c** Mitochondrial morphology was observed by TEM in T24, 5637, and UM-UC-3 cells following Parkin overexpression, scale bar: 1 μm, 200 nm (enlarged). **d** Representative immunofluorescence images of GFP-Parkin overexpressed in T24 cells, labeled with control and CCCP-treated mitochondria (MitoTracker), are shown. *n* = 5, scale bar: 20 μm. Representative immunofluorescence images of 5637 (**e**) and UM-UC-3 (**f**) cells stably transfected with GFP-LC3, overexpressing Flag-Parkin, and labeled with DMSO or CCCP + CQ, are shown. The statistical graphs presented on the right quantify the GFP-LC3 puncta (green) associated with mitochondria (red) in both Parkin-negative and Parkin-positive cells (blue) (scale bar: 20 μm, *n* = 3, one-way ANOVA, a total of 28–32 slides were randomly acquired, with each slide containing a minimum of one Parkin-positive cell). **g** Representative immunofluorescence images of 5637 cells stably transfected with mCherry-EGFP-LC3, overexpressing Flag-Parkin (blue), and labeled with CCCP + CQ, are shown. The corresponding statistical analysis is presented on the right (scale bar: 20 μm, *n* = 3, one-way ANOVA, a total of 52 slides were randomly acquired, with each slide containing a minimum of one Parkin-positive cell). **h** The intracellular ROS levels were measured by DCFH-DA via immunofluorescence in T24 (scale bar: 120 μm, *n* = 5, unpaired two-tailed Student’s *t*-test). The mitochondria ROS levels (**i**) and mitochondria H_2_O_2_ (**j**) were measured by MitoSOX Red and MitoPY1 via immunofluorescence in T24 (scale bar: 120 μm, *n* = 5, unpaired two-tailed Student’s *t*-test). **k** The proportion of apoptotic 5637 cells was determined by flow cytometry after overexpression of Parkin (*n* = 3, unpaired two-tailed Student’s *t*-test). **l** Cell cycle analysis of T24 cells transfected with empty vector or Parkin was performed by flow cytometry, with or without the addition of NAC. *n* = 3, one-way ANOVA. Data are the mean ± SD. Exact *p* values are shown. The *n* number represents *n* biologically independent experiments in each group.

Parkin is an essential molecule in the mitophagy pathway that transmits signals during the activation of autophagy^8,46^. However, recent studies have shown that Parkin also plays roles independent of autophagy^9^. Here, we aimed to investigate whether the effects of Parkin on BLCA are linked to autophagy. First, we used the JC-1 assay kit to determine the level of membrane potential after Parkin overexpression and found no significant difference in the membrane potential depolarization ratio in T24, 5637, and UM-UC-3 cells (Fig. 3a and Supplementary Fig. 3a). Next, we measured SQSTM1/p62 and protein changes reflecting changes in LC3-II and LC3-I during autophagy and found that Parkin overexpression was not related to autophagy-related proteins (Fig. 3b). Using transmission electron microscopy (TEM), we observed the morphology and quality of mitochondria after Parkin overexpression and found that mitochondrial morphology and quality were normal in T24, 5637, and UM-UC-3 cells (Fig. 3c). We further carried out multiple immunofluorescence experiments to investigate this phenomenon. First, following transient transfection of GFP-Parkin plasmids in BLCA cells, we labeled the cell mitochondria with MitoTracker and observed the localization of Parkin under basal conditions and after inducing mitophagy with CCCP via confocal microscopy. The results revealed that in T24 cells, transient transfection of GFP-Parkin plasmids resulted in a uniform distribution of Parkin in the cytoplasm and nucleus (Fig. 3d). However, upon mitophagy induction with CCCP, Parkin significantly aggregated and accumulated on mitochondria (Fig. 3d). This phenomenon was further confirmed in 5637 and UM-UC-3 cells (Supplementary Fig. 3b, c). Considering the established role of Parkin in mitophagy, we infer that in human BLCA cells, mitophagy activation leads to the activation and enrichment of Parkin on mitochondria, thereby participating in subsequent mitophagy processes—a classic function of Parkin in mitophagy. Additionally, we stably transfected 5637 and UM-UC-3 cells with GFP-LC3 lentivirus and subsequently transiently transfected them with Flag-Parkin plasmids (Fig. 3e, f). After treatment with CQ for 8 h followed by CCCP for 4 h, we stained the mitochondria of stably transfected GFP-LC3 cells using MitoTracker. Subsequently, the fixed cells were subjected to immunofluorescence and imaging experiments. The results demonstrated that, in the control group, there were no or few autophagosomes (GFP-LC3 puncta, Fig. 3e, f). The addition of the autophagy inhibitor CQ inhibited the binding of autophagosomes to lysosomes; further stimulation by CCCP activated mitophagy and increased the number of autophagosomes (GFP-LC3 puncta, Fig. 3e, f). Additionally, compared to Parkin-negative cells, both stable GFP-LC3 lentivirus-transfected 5637 and UM-UC-3 cells expressing Parkin accumulated more autophagosomes (GFP-LC3 puncta, Fig. 3e, f). Furthermore, we constructed stable mCherry-EGFP-LC3 lentivirus-transfected 5637 cell lines following similar procedures as above. The results indicated that upon treatment with CQ and CCCP stimulation in mCherry-EGFP-LC3 lentivirus-transfected stable 5637 cell lines expressing Parkin accumulated more autophagosomes (labeled in yellow by mCherry-EGFP-LC3, Fig. 3g). These findings demonstrated that upon activation of mitophagy in bladder cancer cells, the presence of Parkin leads to its aggregation on mitochondria, recruiting more autophagosomes and thereby accelerating mitophagy processes. Finally, we provided further evidence of consistent protein levels of LC3I/II in cells with stable Parkin expression, irrespective of whether Parkin was overexpressed (Fig. 3b and Supplementary Fig. 3d). However, upon the addition of CCCP and CQ, Parkin-overexpressing cells exhibited elevated levels of LC3II protein (Supplementary Fig. 3d). Subsequently, we observed heightened intracellular levels of ROS and unchanged levels of mitochondrial ROS (mROS) and mitochondrial hydrogen peroxide (mH_2_O_2_) in T24, 5637, and UM-UC-3 cells following Parkin overexpression, as evidenced by immunofluorescence (Fig. 3h-j and Supplementary Fig. 3e–g) and flow cytometry analysis (Supplementary Fig. 4a–c), in comparison to the control cells. To examine whether the inhibition of BLCA cell proliferation and migration following Parkin overexpression was caused by ROS, we added 10 mM NAC (N-Acetyl-L-cysteine, a ROS inhibitor) or 1 mM H_2_O_2_ solution to the empty vector- and Parkin-overexpressing cells. After 24 h, a clonogenic assay was performed, and we observed that NAC restored cell proliferation inhibited by Parkin overexpression, while H_2_O_2_ deepened the inhibitory effect on cells (Supplementary Fig. 4d). Since ROS may cause cells to undergo apoptosis and Parkin can affect apoptosis by phosphorylating the BCL-2 protein^47^, we examined the apoptotic changes in 5637 cells after Parkin overexpression. Flow cytometry results showed that overexpression of Parkin failed to increase the proportion of apoptotic cells (Fig. 3k). In light of reports suggesting that ROS may influence cell cycle arrest and subsequently impact cell proliferation^48^, we proceeded to conduct cell cycle analysis on cells overexpressing Parkin. These findings revealed a partial S-phase arrest in T24 and UM-UC-3 cells, which was mitigated by NAC treatment (Fig. 3l and Supplementary Fig. 3h).

### Parkin negatively regulates Catalase degradation by ubiquitination

**Fig. 4:**
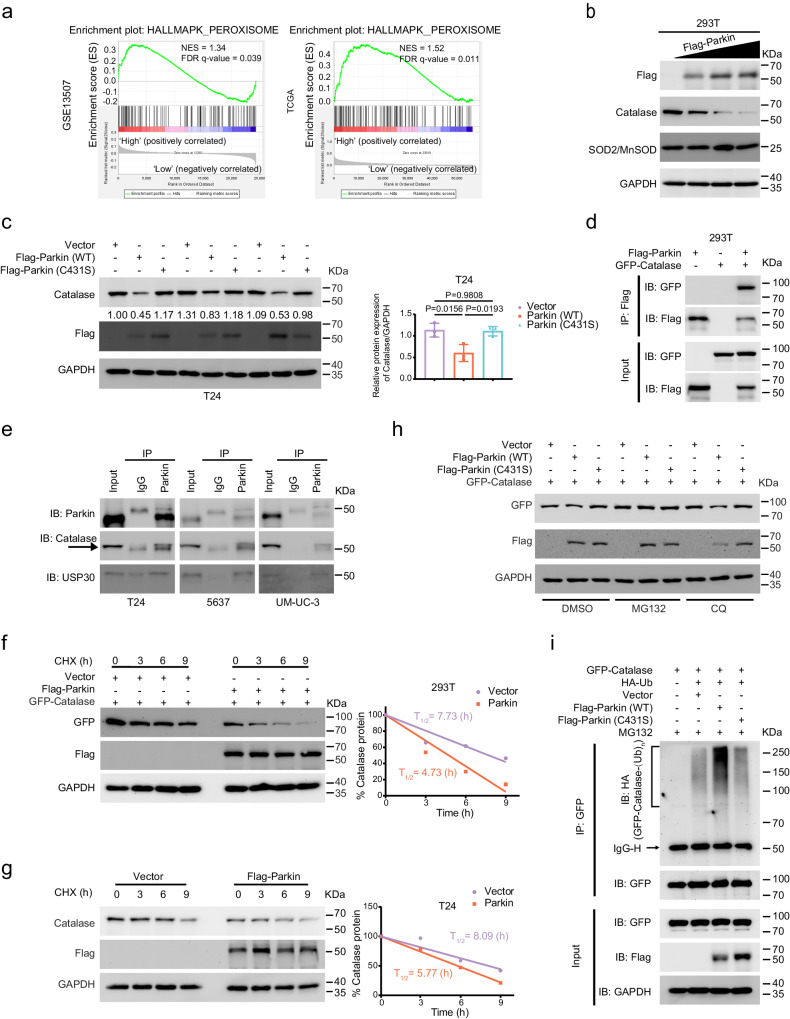
Parkin and Catalase were negatively correlated and interacted. **a** Gene set enrichment analysis (GSEA) was performed via the expression level of *PRKN* in the GSE13507 dataset (left) and TCGA database (right). FDR, false discovery rate; NES, normalized enrichment score. **b** Western blot shows Catalase protein changes after dose-dependent upregulation of Parkin in 293 T cells. **c** Western blot analysis showed protein changes in Catalase after Parkin and Parkin (C431S) overexpression in T24 cells. The corresponding statistical analysis is presented on the right (*n* = 3, one-way ANOVA) **d** Exogenous Parkin protein and Catalase protein were detected in 293 T cells by co-IP assay. **e** Endogenous co-IP assays revealed the concomitant presence of Parkin, USP30, and Catalase proteins in T24, 5637, and UM-UC-3 cells stably expressing Parkin. The protein bands are indicated by arrow symbols for Catalase. **f** Western blot analysis showing associated protein levels after the addition of CHX at different times in 293 T cells overexpressing Parkin. **g** Western blot analysis of Parkin-overexpressing T24 cells after the addition of CHX for different durations. **h** Western blot showing the protein levels of GFP-Catalase after treatment with DMSO, MG132, or CQ in 293 T cells overexpressing Parkin and Parkin (C431S). **i** An in vivo ubiquitination assay of Parkin overexpression was performed in 293 T cells. Data are the mean ± SD. Exact *p* values are shown. The *n* number represents *n* biologically independent experiments in each group.

Initially, to investigate the link between Parkin upregulation and increased intracellular ROS levels, we performed GSEA on the TCGA database and GSE13507 dataset. Our results showed that the high-Parkin group was highly related to peroxidase (Fig. 4a). We then examined several redox proteins by Western blot and found that Catalase, but not SOD2/MnSOD, decreased with increased Parkin protein expression (Fig. 4b). To verify whether this regulatory mechanism was present in BLCA cells, we transfected empty vector, Parkin, and Parkin loss-of-function mutant (C431S) plasmids into T24, 5637, and UM-UC-3 cells and found that Parkin indeed negatively regulated Catalase (Fig. 4c and Supplementary Fig. 5a). However, transcription levels did not change in T24, 5637, or UM-UC-3 cells (Supplementary Fig. 5b). To ascertain whether this regulation occurs through direct interaction or indirect effects, we conducted co-immunoprecipitation (co-IP) experiments in 293 T cells and bladder cancer cells. Moreover, we found that the two proteins interacted by co-IP in 293 T cells, while Parkin (C431S) reduced this effect (Fig. 4d and Supplementary Fig. 5c). In addition, endogenous IP in Parkin-overexpressing BLCA cells revealed a interaction between Parkin and endogenous Catalase (Fig. 4e). Given the crucial role of Parkin as an E3 ubiquitin ligase and its demonstrated interaction, we posited that Catalase could be a substrate of Parkin. To explore whether Parkin influences the stability of Catalase, we conducted cycloheximide (CHX) assays. Our observations revealed a significant reduction in the half-life of Catalase following Parkin overexpression in both 293 T and BLCA cells (Fig. 4f, g). To investigate the mechanism of Catalase degradation, we added DMSO (as a control), MG132 (10 μM, a proteasome inhibitor), or CQ (10 μM, an autophagy lysosome inhibitor). Western blot results showed that MG132, but not CQ, effectively restored the reduced Catalase induced by Parkin overexpression (Fig. 4h), which was further confirmed in Parkin BLCA cells (Supplementary Fig. 5d). Thus, the Parkin-induced reduction in Catalase depends on the proteasome pathway. Since Parkin acts as an E3 ubiquitin ligase, we hypothesized that Catalase may be specifically recognized by Parkin and undergo ubiquitin-mediated degradation. Western blot analysis confirmed that the ubiquitination level of Catalase increased significantly during Parkin overexpression (Fig. 4i), supporting our hypothesis.

### CAT knockdown inhibited the proliferation of BLCA cells, while overexpression had the opposite effect

**Fig. 5:**
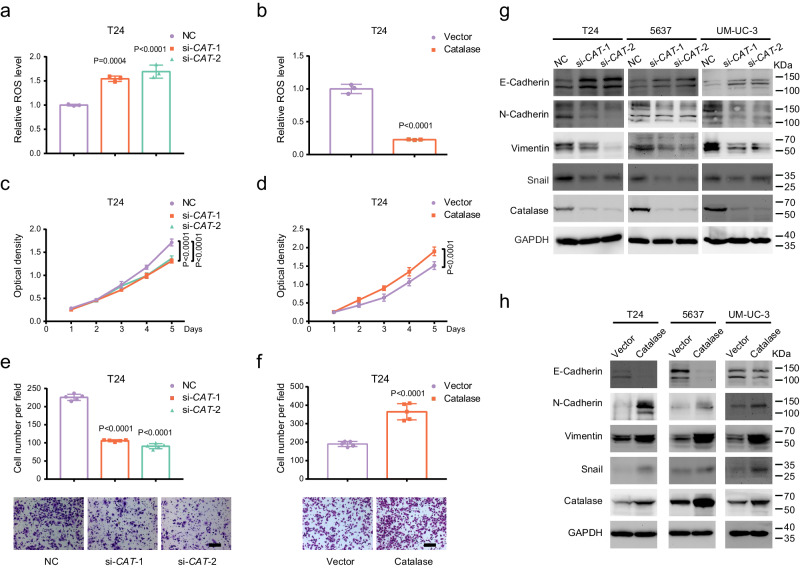
Catalase upregulation promotes cell proliferation and migration, and *CAT* knockdown has the opposite effect. The intracellular ROS levels were measured after *CAT* knockdown (**a**) (*n* = 3, one-way ANOVA) and Catalase overexpression (**b**) (*n* = 3, unpaired two-tailed Student’s *t*-test) by DCFH-DA via flow cytometry in T24 cells. The MTT assay indicates the proliferative capacity of T24 cells after *CAT* knockdown (**c**) and overexpressing Catalase (**d**) (*n* = 8, two-way ANOVA). Transwell assays represent the migration capacity of T24 cells after knockdown of *CAT* (**e**) (scale bar: 200 μm, *n* = 5, one-way ANOVA) and Catalase overexpression (**f**) (scale bar: 200 μm, *n* = 5, unpaired two-tailed Student’s *t*-test). Western blot indicates the expression of EMT-related proteins after knockdown of *CAT* (**g**) and Catalase overexpression (**h**) in BLCA cells. Data are the mean ± SD. Exact *p* values are shown. The *n* number represents *n* biologically independent experiments in each group.

To evaluate Catalase’s potential role in human BLCA, we knocked down and overexpressed Catalase in BLCA cells and verified its efficiency (Supplementary Fig. 6a-b). As an important oxidoreductase, Catalase decomposes excess H_2_O_2_ in cells into H_2_O and O_2_, leading to a decrease in ROS levels in cells^49^. Therefore, we first examined the ROS level in the cells by flow cytometry and found that ROS levels significantly increased after *CAT* knockdown in T24, 5637, and UM-UC-3 cells (Fig. 5a, Supplementary Fig. 6c, e and g), whereas overexpression showed the opposite effect (Fig. 5b, Supplementary Fig. 6d, f, and h). Next, we evaluated cell proliferation using MTT and clonogenic assays, which showed that *CAT* deficiency evidently attenuated proliferation in T24, 5637, and UM-UC-3 cells (Fig. 5c, Supplementary Fig. 6i, k, m, 7a and c), whereas Catalase overexpression significantly increased proliferation in T24, 5637 and UM-UC-3 cells (Fig. 5d, Supplementary Fig. 6j, l, n, 7b and 7d). We then evaluated the effect of Catalase on the migratory ability of tumor cells by Transwell and wound healing assays. Silencing *CAT* markedly decreased the migratory capacity in T24, 5637, and UM-UC-3 cells (Fig. 5e, Supplementary Fig. 7e, g, i, and k), and overexpression of Catalase distinctly enhanced the migratory capacity in T24, 5637, and UM-UC-3 cells (Fig. 5f, Supplementary Fig. 7f, h, j and l). The Western blot results showed that knockdown of *CAT* could decrease the generation of mesenchymal-derived proteins such as N-Cadherin, Snail, and Vimentin during EMT but increase epithelial-derived E-Cadherin (Fig. 5g), while overexpression had the opposite effects (Fig. 5h). The above results indicate that Catalase regulates the intracellular ROS level in BLCA cells and functions as an oncogene in BLCA by causing changes in tumor cell phenotypes.

### USP30 can be negatively regulated by Parkin and deubiquitinated to stabilize Catalase

**Fig. 6:**
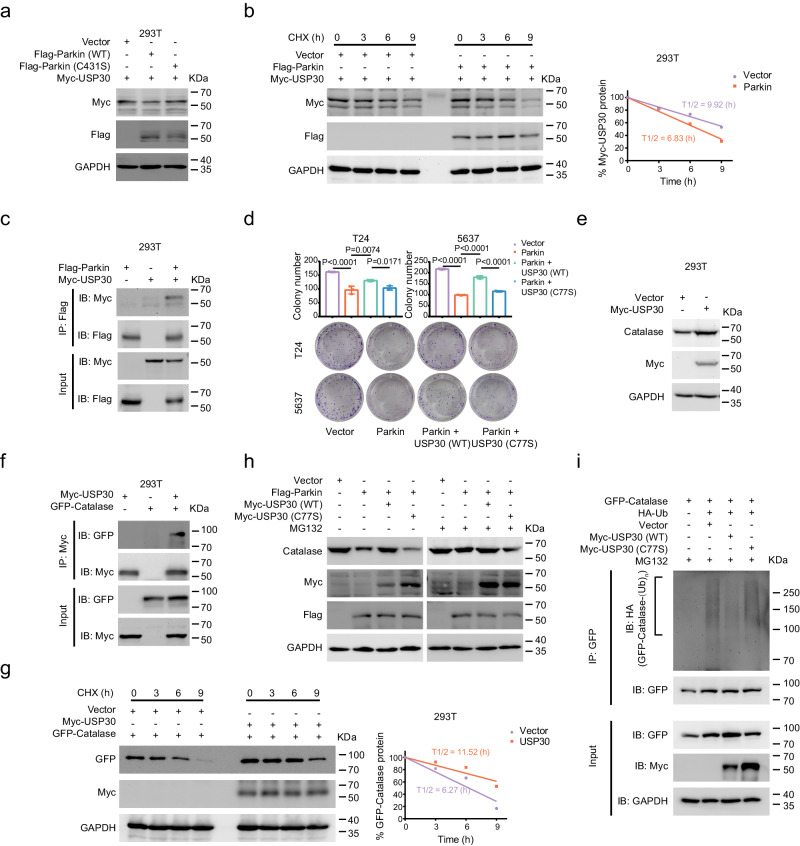
USP30 interacts with Catalase and stabilizes Catalase by deubiquitination. **a** Western blot showing Myc-USP30 protein expression after overexpression of Parkin (WT) and Parkin (C431S) in 293 T cells. **b** Western blot showing the relative percentage of Myc-USP30 protein expression at different time points after the addition of CHX to 293 T cells overexpressing Parkin. **c** Flag-Parkin and Myc-USP30 proteins were detected in 293 T cells by co-IP assay. **d** A clonogenic assay was used to evaluate cell viability after overexpressing USP30 and USP30 (C77S) in stable Parkin-overexpressing T24 and 5637 cells (*n* = 3, one-way ANOVA). **e** Western blot showing endogenous Catalase protein expression after overexpression of USP30 in 293 T cells. **f** Myc-USP30 and GFP-Catalase proteins were evaluated in 293 T cells by co-IP assay. **g** Western blot showing the relative percentage of 293 T cells with USP30 overexpression expressing the GFP-Catalase protein at different time points after the addition of CHX. **h** Western blot showing changes in the protein levels of endogenous Catalase after the overexpression of Parkin, USP30 or USP30 (C77S) in 293 T cells with DMSO or MG132. **i** An in vivo ubiquitination assay of USP30 overexpression was performed in 293 T cells. Data are the mean ± SD. Exact *p* values are shown. The n number represents n biologically independent experiments in each group.

Several studies have reported that USP30 is a classic antagonist of Parkin^40,50,51^. Bingol et al. demonstrated that USP30 can be ubiquitinated by Parkin and act as a substrate^51^. Here, we first found that the overexpression of Parkin, rather than Parkin (C431S), could reduce the protein level of USP30 (Fig. 6a). A CHX assay showed that overexpression of Parkin strongly decreased the half-life of the exogenous USP30 protein (Fig. 6b). We further confirmed the interaction between Parkin and USP30 through co-IP (Fig. 6c). Although we validated this interaction and established the existence of mutual interaction between Parkin and USP30 in BLCA cells (Fig. 4e), it remains unclear whether USP30 is involved in the Parkin-mediated regulation of catalase in bladder cancer. To investigate whether the regulation of Catalase by Parkin is related to USP30 in BLCA cells, we transfected empty vector, USP30, and USP30 (C77S, a common point mutation affecting enzyme activity) into stable Parkin-overexpressing T24 and 5637 cells. We found that the addition of USP30, but not USP30 (C77S), partially restored the growth-inhibiting effect of Parkin in the cells (Fig. 6d). Next, we investigated how USP30 affects Parkin-mediated regulation of Catalase. First, USP30 has been implicated in peroxisomal autophagy^41,44,45,52^. Given that catalase is one of the major proteins in peroxisomes, we investigated the potential impact of USP30, a deubiquitinating enzyme, on Catalase. We found that overexpression of USP30 could directly increase endogenous Catalase (Fig. 6e) and that USP30 interacted with Catalase (Fig. 6f). Given the significance of USP30 as a deubiquitinating enzyme and its demonstrated interaction with Catalase, we hypothesized that Catalase could be a substrate of USP30. To explore whether USP30 influences the stability of Catalase, we conducted CHX assay. CHX assay showed that, compared with empty vector, the overexpression of USP30 strongly increased the half-life of the exogenous Catalase protein (Fig. 6g). To further elucidate the mechanism of Catalase stabilization, we treated cells with DMSO or MG132 in our investigation. Among the endogenous Catalase, only USP30, not USP30 (C77S), exhibited increased expression (Fig. 6h). The effect of USP30 on Catalase was found to be dependent on the proteasome pathway, as MG132 inhibited proteasomal degradation and amplified the positive regulatory effect of USP30 on Catalase (Fig. 6h). Moreover, Western blot analysis of 293 T cells revealed that the level of ubiquitinated Catalase was lower in cells overexpressing wild-type USP30 than in those in the empty vector group (Fig. 6i), indicating that USP30 can stabilize Catalase by deubiquitinating it. Taken together, these findings suggest that USP30 plays a critical role in stabilizing Catalase via deubiquitination and is involved in the regulation of Catalase by Parkin in BLCA cells.

### Parkin inhibits the proliferation and migration of BLCA cells via Catalase

**Fig. 7:**
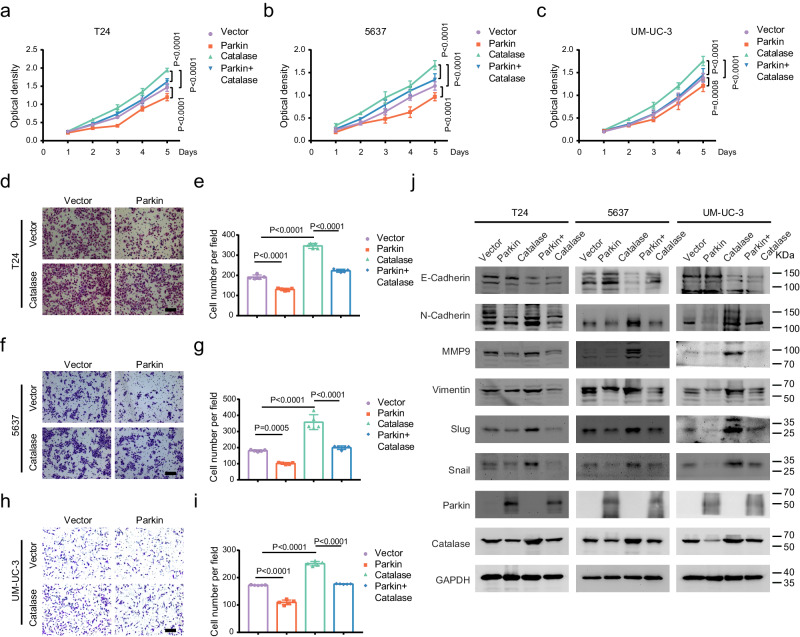
Parkin inhibits proliferation and migration by Catalase. MTT assays showing changes in the proliferation of T24 (**a**), 5637 (**b**), and UM-UC-3 (**c**) cells after overexpressing empty vector, Parkin, Catalase, and Parkin + Catalase (*n* = 8, two-way ANOVA). Transwell assays represent the migration capacity of cells after overexpression of empty vector, Parkin, Catalase, and Parkin + Catalase in T24 (**d**, **e**), 5637 (**f**, **g**), and UM-UC-3 (**h**, **i**) cells (scale bar: 200 μm, *n* = 5, one-way ANOVA). **j** Western blot analysis indicated changes in EMT-related proteins after overexpression of empty vector, Parkin, Catalase, and Parkin + Catalase in BLCA cells. Data are the mean ± SD. Exact *p* values are shown. The *n* number represents *n* biologically independent experiments in each group.

To ascertain the combined effects of Parkin and Catalase in BLCA, we divided the cells into four groups for subsequent cell function experiments. First, we examined the intracellular ROS levels in transiently transfected cell lines. We found that overexpression of Parkin increased ROS levels, while overexpression of Catalase decreased ROS levels (Supplementary Fig. 8a–c). The altered ROS levels were partially restored when both plasmids were transfected together (Supplementary Fig. 8a-c). Then, we generated stable lentiviral cells (empty vector, Parkin, Catalase, and Parkin + Catalase) and evaluated cell proliferation using MTT and clonogenic assays. We found that overexpression of Catalase rescued the Parkin-mediated inhibition of cell proliferation in T24, 5637, and UM-UC-3 cells (Fig. 7a–c and Supplementary Fig. 8d–f). Next, we determined the effect on tumor cell migratory ability using Transwell and wound healing assays and found that overexpression of Catalase rescued the Parkin-mediated inhibition of cell migration in T24, 5637, and UM-UC-3 cells (Fig. 7d–i and and Supplementary Fig. 8g–i). Western blot analysis showed that Catalase overexpression rescued the expression of mesenchymal-derived proteins such as N-Cadherin, MMP9, Slug, Snail, and Vimentin, which were inhibited by Parkin overexpression in T24, 5637, and UM-UC-3 cells (Fig. 7j). Furthermore, it reduced the increase in epithelial-derived E-Cadherin induced by Parkin overexpression (Fig. 7j). The results of the above experiments indicate that Parkin inhibits the proliferation and migration of BLCA cells via Catalase.

### Parkin suppresses tumor growth and lung metastasis in vivo

**Fig. 8:**
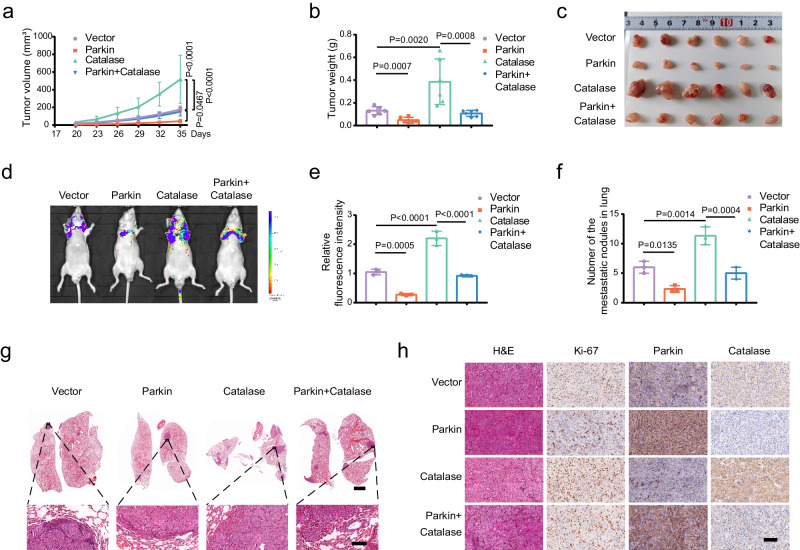
Parkin suppresses tumor growth and lung metastasis in mice by Catalase. **a** The tumor volume was measured at different time points (*n* = 6, two-way ANOVA). **b** The tumor weights of the different groups (*n* = 6, one-way ANOVA). **(c)** The subcutaneous tumors were surgically excised and photographed in different experimental groups (*n* = 6). **d** The fluorescence changes in the lungs were photographed using a small animal intravital imager. **e** Relative quantification of lung fluorescence in (D) (*n* = 3, one-way ANOVA). **f** The number of pulmonary nodules from three mice in each group (*n* = 3, one-way ANOVA). **g** Representative images of H&E staining of the lungs from different groups, scale bar: 2 mm, 100 μm (enlarged). **h** Representative visual field images of H&E and IHC (Ki-67, Parkin, and Catalase) of tumor tissue from different groups, scale bar: 100 μm. Data are the mean ± SD. Exact *p* values are shown. The n number represents n biologically independent experiments in each group.

Next, we used four types of stably transfected lentiviral T24 cell lines (empty vector, Parkin, Catalase, and Parkin + Catalase) to establish xenograft models. Parkin overexpression significantly reduced tumor volume and weight compared to controls. Conversely, Catalase overexpression significantly increased these parameters (Fig. 8a–c). The double-overexpression group then exhibited restored inhibition of tumor growth caused by overexpressing Parkin (Fig. 8a–c). IHC staining revealed that the number of Ki-67 protein-positive cells was lower in the Parkin group than in the control group but was higher in the Catalase group, and the Catalase protein-positive level was extremely low in the Parkin group (Fig. 8h). Additionally, by injecting four groups of cells (1 × 10^5^/50 μL) into the tail vein and imaged after 50 days, we found that overexpression of Parkin inhibited the lung metastasis of tumor cells in mice (Fig. 8d, e), while overexpression of Catalase promoted lung metastasis (Fig. 8d, e). The simultaneous overexpression of Parkin and Catalase significantly restored the lung metastatic capacity of tumor cells in mice overexpressing Parkin (Fig. 8d, e). Furthermore, the number of pulmonary nodules in each group was consistent with the above changes (Fig. 8f, g). The results of the above experiments showed that Parkin inhibited the tumorigenicity and lung metastasis ability of BLCA cells by inhibiting Catalase in mice.

## Discussion

Parkin was initially discovered in Parkinson’s disease^7^ and subsequently identified as a key molecule in mitophagy^53^. Mitophagy removes damaged mitochondria, thereby exerting neuroprotective effects in multiple studies^8,51^. Several reviews have indicated a link between Parkinson’s disease and cancer^54,55^, including lung cancer^11,56^, melanoma^57^, and glioblastoma^58^. Additionally, mitophagy plays a role in cancer regulation under specific conditions^12,13,59,60^. This raises the question of whether Parkin plays an important yet unknown role in cancer. Indeed, recent years have seen a growing number of studies examining Parkin’s role in various cancers^9–11^, which indicate lower levels and poor prognosis in multiple cancers^17,18,56^. Therefore, we aimed to investigate Parkin’s function in BLCA.

**Fig. 9:**
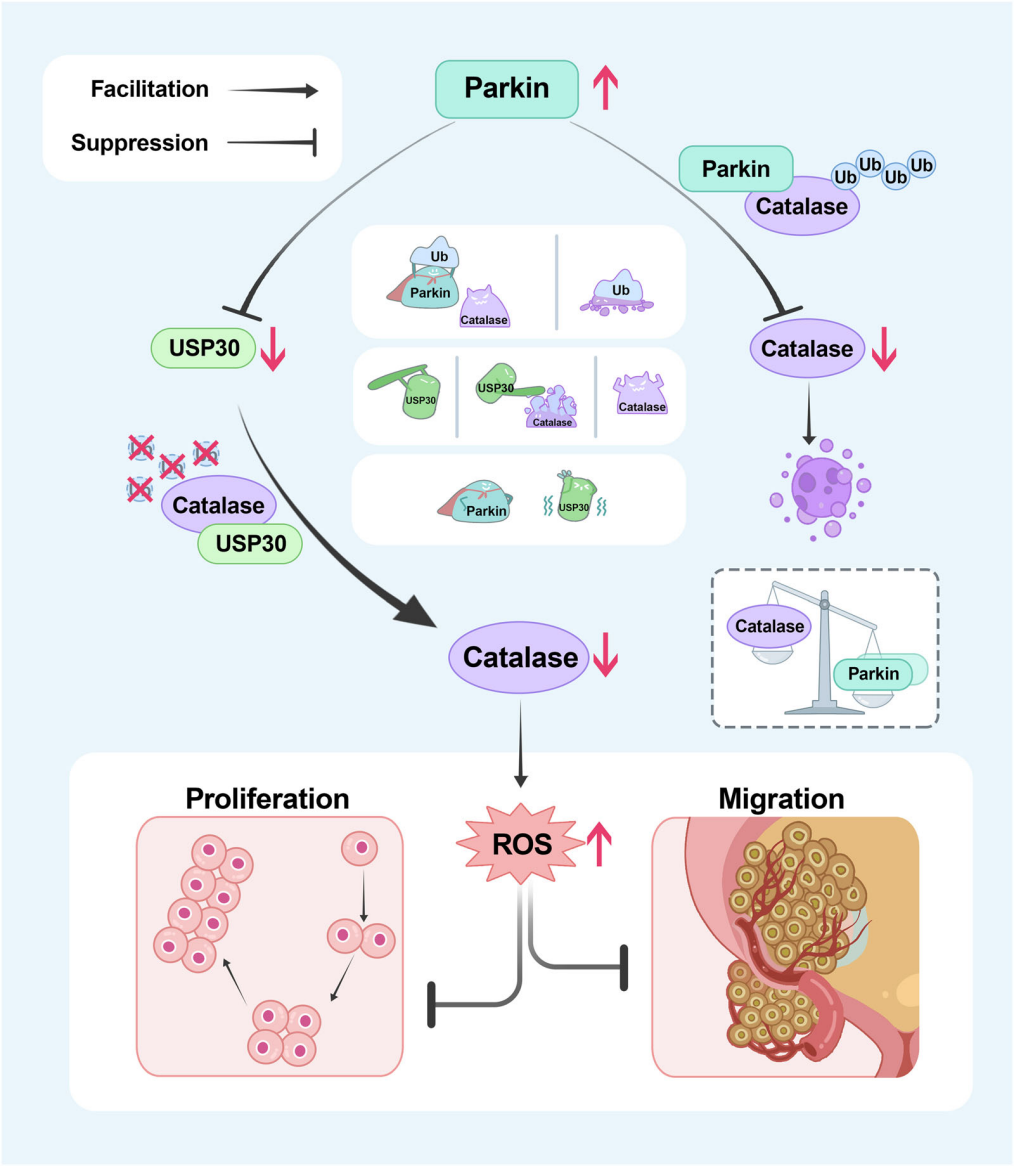
Mechanistic diagram of this study. This study elucidates that Parkin negatively regulates Catalase, leading to an increase in intracellular ROS levels and consequently impeding the proliferation and migration of BLCA cells. Mechanistically, Parkin directly decreases the protein level of Catalase through ubiquitination. Moreover, Parkin degrades the established deubiquitinating enzyme USP30, thereby diminishing its stabilizing effect on catalase through deubiquitination, ultimately resulting in an indirect reduction in the protein level of Catalase. Creation of the illustrastions and every element in Fig. 9 was drafted by the authors, and edited by Dr. Yuruo Chen, a diagram editing expert at the Chinese Academy of Science using Adobe Photoshop software. No artificial intelligence or database was involved in the creation of this image.

We examined a variety of BLCA data sets, and combined with human tissue microarray (TMA), we found that Parkin is expressed at lower levels in cancer tissues, which is consistent with previous studies^17,18,56^. We transfected BLCA cells with either transient or stable Parkin and observed significant inhibition of cell proliferation and migration. Furthermore, intracellular ROS, rather than mitochondrial ROS or mitochondrial H_2_O_2_, influence this phenomenon. Additionally, Parkin-dependent autophagy did not occur during this process, indicating that this phenotypic change was not dependent on the mitophagy pathway. Moreover, published reports suggest that only ROS originating from mitochondria can induce Parkin recruitment and initiate Parkin-dependent mitochondrial degradation^61^. Another study reported that the primary component of ROS, superoxide, rather than hydrogen peroxide, serves as the major regulator of Parkin/PINK1-dependent mitophagy^62^. Emerging functions of Parkin in cancer, including metabolic reprogramming^9^, serine synthesis^10^, and necrotizing apoptosis^15^, have also been reported and could be attributed to its E3 ligase activity even without mitophagy. Further studies demonstrated that Parkin can regulate intracellular ROS levels by modulating the expression of Catalase, a protein related to redox reactions^25^. Mechanistic studies demonstrated that Parkin interacts with Catalase and negatively regulates it by ubiquitination. In addition, we found that USP30, an inhibitor of Parkin, also participated in the process of inhibiting BLCA cell proliferation^41^. USP30 was also found to be a substrate of Parkin^51^. We further revealed that USP30 can positively regulate Catalase, and speculated that the regulation of Catalase by Parkin could also be regulated by the intermediate protein USP30. Our results confirmed our speculation that Parkin indirectly regulates Catalase by reducing its stabilization through USP30. A diagram of the underlying mechanism is shown in Fig. 9.

In the course of activated mitophagy facilitated by Parkin, the latter serves as a crucial mitophagy factor that effectively eradicates impaired mitochondria and their associated proteins, consequently mitigating the levels of ROS within the mitochondria^63^, although its effect may differ on certain occasions^9^. A study found that consuming Parkin could attenuate PD toxin-induced H_2_O_2_^64^_._ Some research indicates that Parkin may play different roles in various intracellular environments, but they all stem from its enzymatic activity. Various studies have explored the ubiquitination of Parkin^9,65–68^. For instance, by using induced neurons from embryonic stem cells and performing quantitative proteomics, one study demonstrated the potential specificity of the central neural regulation of Parkin-modified ubiquitin^68^. Another study utilized comparative genomics and protein domain mapping analysis to identify novel functional relationships between Parkin ubiquitination and RNA metabolism in proteomes^67^. Additionally, Rose et al. reported a cell line and tissue sample-compatible method to reveal the protein and ubiquitination status of mitochondria undergoing PINK1/Parkin mitophagy, identifying an extensive collection of targets ubiquitinated by Parkin and PINK1^66^. All these studies demonstrate the critical and unique role of Parkin in ubiquitin-mediated degradation.

High levels of oxidative stress are common characteristics of several diseases, including tumors. In recent years, several new types of cancer cell death, such as ferroptosis, cuproptosis, disulfidocytosis, and necrotic apoptosis, have been discovered in tumors. These types of cell death alter intracellular oxidative stress and create barriers to the synthesis or transport of essential substances, resulting in the accumulation of toxic substances that ultimately lead to high levels of ROS. These ROS can damage proteins, DNA, and lipids. Several studies have found a correlation between Parkin and multiple biological phenomena, including lactate metabolism, serine metabolism, necrotic apoptosis, immunometabolism, and lipid remodeling. Parkin may be reminiscent of other cardinal tumor suppressors, such as p53, suggesting that the two proteins are highly similar in many aspects. Therefore, Parkin may be considered the next “p53”^9^.

This study has several limitations that should be noted. First, the low or absent expression levels of Parkin in many BLCA cell lines make it difficult to observe the biological phenomena of cells effectively when *PRKN* is knocked down or knocked out. Second, our study found that the effects of Parkin on BLCA are independent of mitophagy. Specifically, within BLCA cells, the expression of Parkin does not induce autophagy, as indicated by the expression of LC3. However, Parkin is still capable of engaging in mitophagy when cells receive specific signals. Furthermore, the overexpression of Parkin results in elevated levels of ROS; nevertheless, this overexpression does not impact mitochondrial ROS and mitochondrial H_2_O_2_ levels. While Parkin primarily functions to recruit autophagy-related proteins and degrade them to reduce ROS levels within mitochondria upon activation of mitophagy, its role may vary in the basal state. Additionally, ROS can act as an important signal to activate mitophagy, but the quantity levels of ROS and the balance between oxidized and reduced proteins in different cells and microenvironments are also important factors that can determine downstream signaling pathways.

In summary, our study uncovers a previously unknown role of Parkin in cancer cells under oxidative stress without inducing mitophagy. Specifically, we demonstrate that Parkin targets antioxidant proteins to suppress tumor growth and metastasis through a pathway that does not involve mitophagy activation. We also show that Parkin’s E3 ligase activity is necessary for targeting the key redox protein Catalase for degradation. Furthermore, we demonstrate that USP30, a known Parkin substrate, can deubiquitinate and stabilize Catalase. These mechanisms lead to altered intracellular ROS levels and induce cell cycle arrest, ultimately inhibiting bladder cell proliferation and reducing metastatic tumor growth and lung metastatic capacity in vivo.

## Methods

### Human tissue samples

The study using human bladder cancer tissues and paired paracancerous tissues (*n* = 9) was approved by the Institutional Ethics Committee of Zhongnan Hospital of Wuhan University (approval number: 2021125). Informed consent was obtained from all individuals. The bladder cancer and paired paracancerous tissues were collected after radical bladder cancer surgery and pathological confirmation.

A human tissue microarray (TMA) consisting of 63 patients with confirmed BLCA (including 16 adjacent tissues) and clinical characteristics was purchased from Shanghai Outdo Biotech. Clinical information is provided in Supplementary Table 1, and immunohistochemical (IHC) staining was performed using an anti-Parkin antibody. The analysis process was blinded to the clinical outcomes, clinical characteristics, and pathological stages. Tissue sections were scanned and imaged using a slice scanner. The TMA plug-in in Quant Center 2.3 analysis software was used to set the chip tissue point diameter size, rank the number and generate the number automatically. Positive grades were assigned using the Densito quant module in the Quant Center 2.3 analysis software: negative with no coloring, weak positive light yellow, medium positive light yellow and strong positive tan were counted as “0”, “1”, “2” and “3” points, respectively. The number of weak, medium, and strong positive cells and the total cell number were calculated in the measured area. The percentage of positive cells and the positive score per spot were calculated from the number of positive cells (percentage: 1 < 25%, 2 = 25-50%, 3 = 50-75%, 4 > 75%). The staining signal in the tumor cells was quantified using a scoring system of 0 to 12. The final score was obtained by multiplying the positive grade by the percentage of positive cells. Low and high expression were defined as scores < average and ≥ average, respectively.

### Cell culture

HEK293T cells and the human BLCA cell lines SV-HUC-1, T24, UM-UC-3, RT4, 5637, ScaBER and J82 were acquired from the Shanghai Cell Bank (Chinese Academy of Sciences). HEK293T cells were cultured in DMEM (HyClone), while SV-HUC-1, T24, RT4, 5637, ScaBER and J82 cells were cultured in RPMI-1640 (Gibco), and UM-UC-3 cells were cultured in MEM (Gibco). The cell lines described above were cultured in 10% FBS-supplemented medium (ExcellBio, FSP500) and verified using short tandem repeat (STR) assays. Mycoplasma was not detected in any of the cell lines.

### Plasmids and transfection

The *PRKN* cDNA was ligated into the pcDNA3.1-3×Flag vector. The plasmid GFP-Parkin was obtained from MiaoLing Plasmid Platform (Wuhan, China). The USP30 cDNA was inserted into the pEnCMV-3×Myc vector, while the *CAT* cDNA was inserted into the pcDNA5 and pCMV-EGFP vectors. All the plasmids were sequenced prior to use to ensure the accuracy of their sequences. The small interfering RNA (siRNA) against *CAT* was purchased from GenePharma, and the sequence information was as follows: si-CAT-1, sense, 5’-CCAAAUACUCCAAGGCAAATT-3’; si-CAT-2, sense, 5’-GGAAACGUCUGUGUGAGAATT-3’. The recombinant plasmids were sequenced to confirm the absence of sense mutations. Parkin (C431S) and USP30 (C77S) point mutant plasmids were constructed using site-directed mutagenesis kits and verified by DNA sequencing. The cells were transiently transfected into cells with Lipofectamine 3000 (Invitrogen). The LV5-NC, LV8N-NC, LV5-CAT, LV8N-Parkin, GFP-LC3, and mCherry-EGFP-LC3 lentiviruses were purchased from GenePharma.

### RNA extraction, reverse transcription and quantitative reverse transcription PCR (qRT-PCR)

Total RNA from 1×10^5^ cells was first isolated using an RNA extraction kit (R4111-03, Magen) according to the manufacturer’s instructions. cDNA was obtained using a reverse transcription kit (TOYOBO, FSQ-101) and subjected to qRT-PCR. The sequences of primers used were as follows: *PRKN*-F: 5’-GTGCAGAGACCGTGGAGAAA-3’; *PRKN*-R: 5’-GCTGCACTGTACCCTGAGTT-3’, *CAT*-F: 5’-AAAAGATATCATGGCTGACAGCCGGGAT-3’; *CAT*-R: 5’-AAAAGCGGCCGCTCACAGATTTGCCTTCTC-3’, *GAPDH*-F: 5’-GACTCATGACCACAGTCCATGC-3’; *GAPDH*-R: 5’-AGAGGCAGGGATGATGTTCTG-3’. The CT values of GAPDH were used for normalization.

### Transwell assay

The different groups of cells were seeded at 2–10 × 10^4^/200 μL in a 24-well plate containing a polycarbonate pore filter (Corning). After 16–24 h of incubation, the cells were fixed and stained.

### Wound healing assay

The different groups of cells were placed into plates, and when the cells reached 100% confluence, we scratched the cells with a pipette tip. The cells were incubated in a medium without FBS for 0 or 16-20 h and then photographed with a microscope.

### Clonogenic assay

A total of 1000 cells from different groups were added to six-well plates, and the cells were fixed after 9–12 days of growth and stained.

### MTT assay

Forty-eight hrs after transfection, a total of 3 × 10^3^ BLCA cells were added to 96-well plates for 5 days. After the addition of 20 μL of MTT solution for 4 h, the precipitate was dissolved in 200 μL of DMSO. A spectrophotometer was used to measure the absorbance.

### Flow cytometry

#### Intracellular ROS, mitochondrial ROS (mROS) and mitochondrial H_2_O_2_ (mH_2_O_2_) level

48 h after transfection, a total of 1 × 10^5^ cells were counted and incubated with DCFH-DA (10 μM), MitoSOX Red (10 μM, HY-D1055, MCE) or MitoPY1 (10 μM, #4428, R&D) for 30 mins. The cells were subsequently washed three times in PBS and analyzed on the Cytoflex (Beckman). Intact cells were selected and gated based on the forward scatter/side scatter (FSC/SSC) plot to exclude small fragments.

#### Mitochondrial transmembrane potential (MTP) assay

The membrane potential was determined using the JC-1 detection kit. Briefly, 48 h after transfection, 1 μL of reagent was added, the cells were incubated for 30 mins, and the cells were analyzed on the Cytoflex after three washes. Intact cells were selected and gated based on the forward scatter/side scatter (FSC/SSC) plot to exclude small fragments.

#### Cell apoptosis

For apoptosis analysis, an annexin V-FITC apoptosis kit (Sungene) was used. 48 h after transfection, a total of 1 × 10^5^ transfected 5637 cells were analyzed on the Cytoflex. Intact cells were selected and gated based on the forward scatter/side scatter (FSC/SSC) plot to exclude small fragments.

#### Cell cycle

A total of 1 × 10^5^ transfected T24 and UM-UC-3 cells were collected and washed with cold PBS, followed by centrifugation. The cells were then resuspended in a solution containing propidium iodide (PI, 100 μg/mL) and permeabilization solution from the cell cycle staining kit (CCS012, Multi sciences). After incubating in the dark for 30 mins at room temperature, the cells were analyzed using Cytoflex. FlowJo software (v10.8.1) was employed for result analysis. Intact cells were selected and gated based on the forward scatter/side scatter (FSC/SSC) plot to exclude small fragments.

A figure exemplifying the gating strategy is provided in Supplementary Fig. 9.

### Immunoblotting

The procedure was performed as described previously^69^. Cells were lysed on ice for 30 mins using a mixture of phosphatase inhibitor, protease inhibitor, and RIPA buffer. The supernatant was collected after high-speed centrifugation. The protein concentration was determined using the BCA assay. Protein extracts were separated by SDS-PAGE gel and transferred to PVDF membranes. The membranes were then blocked in TBST buffer containing 5% skim milk and incubated sequentially with primary and secondary antibodies. The proteins were detected using the BioSpectrum 515 Imaging System (UVP), and the primary antibodies used are listed in Supplementary Table 4. In this study, GAPDH served as the only internal control.

### Coimmunoprecipitation (co-IP) assay

Cells were harvested and lysed using mild lysis buffer for half an hour before high-speed centrifugation. One hundred microliters of the supernatant were mixed with 5× loading buffer and boiled for 10 mins at 100°C. The remaining supernatant was incubated with 1 μg of antibody for 16 h, after which 20 μL of fresh protein A/G magnetic beads (BEAVER) was added and incubated for 2 h. The mixture was washed thoroughly. Immunoblotting was performed.

### Cycloheximide (CHX), MG132 and chloroquine (CQ) assays

After 24-48 h, CHX was added at different time points. The cells were treated with 10 μM MG132 or treated with 10 μM CQ for 8 h. Cells were then harvested for Western blot analysis.

### Ubiquitination assay

After transfecting cells for 24-48 h. Cells were then harvested and lysed. One microgram of anti-Catalase antibody was added to the lytic supernatant for immunoprecipitation assays, and the level of Catalase ubiquitination was detected by using an anti-HA antibody.

### IHC staining

The procedure of the above assays was described previously^69^. Briefly, fresh tumors were fixed in 4% PFA for 24 h. Subsequently, the tissues were embedded in paraffin and cut into 5 μm sections. The slides were then sequentially probed with primary and secondary antibodies, as listed in Supplementary Table 4. The DAB chromogen was used for incubation, followed by counterstaining with hematoxylin. Finally, the sections were analyzed under a light microscope.

### Immunofluorescence staining

A total of 1 × 10^5^ cells were seeded in six-well plates containing cell slides. Transfection was performed after 24 h. Before fixation, DMSO was added for 8 h, followed by CQ for 8 h and CCCP for 4 h. DCFH-DA, MitoSOX Red, MitoPY1, and MitoTracker (1:1000, C1032, Beyotime) were added for 10–30 mins. After 24 h, cells were fixed with 4% formaldehyde for 20 mins at room temperature. Subsequently, the cells were washed three times with PBS and incubated with buffer containing 2% BSA and 0.3% Triton X-100 for 1 hr at room temperature. Then, the cells were incubated with the corresponding primary antibody at 4°C for 4 h. After three washes with PBS, the cells were incubated with the secondary antibody for 2 h at room temperature. Following another wash with PBS, the cells were incubated with DAPI (1:1000) for 5 mins at room temperature (Note: Some cells may skip this step and not incubate with DAPI). Finally, the cells were sealed, air-dried overnight, and then photographed using a confocal microscope with a 20× or 60× oil lens.

### Transmission electron microscopy (TEM)

A total of 1 × 10^7^ BLCA cells were transfected and fixed with freshly prepared 2.5% glutaraldehyde at room temperature. The cells were centrifuged at low speed, washed, and fixed in 1% osmic acid for 2 h. The samples were rinsed with 0.1 M phosphate buffer, and different concentrations of ethanol were added to dehydrate the samples. Samples were then infiltrated in 100% acetone: epoxy resin 812 (1:2) and embedded overnight at room temperature. Subsequently, the cells were embedded, cured, and sectioned. Samples were sectioned and then stained with 2% uranyl acetate and lead citrate. Finally, the sections were imaged using an electron microscope (TEM, HT7700, Hitachi) at the Research Center for Medicine and Structural Biology, Wuhan University, China.

### Animal experiments

We purchased 36 male BALB/c nude mice (6 weeks old) from WQJX Bio Technology (Wuhan, China) and adaptively fed them in a specific pathogen-free (SPF) facility for 1 week. We constructed four T24 cell lines (empty vector, Parkin, Catalase, and Parkin + Catalase). For the xenograft model, cells were subcutaneously implanted into nude mice at 1 × 10^6^ cells/150 μL (*n* = 6). We measured tumor length (L) and width (W) every three days and calculated tumor volume (V) using the formula V = 0.5 × L × W^2^. The nude mice were sacrificed for tumor weighing and IHC staining. For the metastasis model, 1 × 10^6^ cells were slowly injected into the tail vein of nude mice (*n* = 3). After 50 days, we measured the lung fluorescence intensity in anesthetized nude mice. The number of lung nodules was counted, and the nodules were fixed in paraformaldehyde for H&E staining. The animal experiments in this study were performed in accordance with the guidelines of the Institutional Experimental Animal Welfare Ethics Committee (approval number: ZN2022242).

### Statistics and reproducibility

The data were analyzed using GraphPad Prism 7. Data are expressed as the mean ± standard deviation (SD). Unpaired two-tailed Student’s t-test, paired two-tailed Student’s *t*-test, the Wilcoxon signed-rank test, Kruskal-Wallis test, one-way ANOVA and two-way ANOVA were used. The n number represents n biologically independent experiments in each group. *p* < 0.05 was considered statistically significant.

### Reporting summary

Further information on research design is available in the Nature Portfolio Reporting Summary linked to this article.

### Supplementary information

Supplementary Information

Description of Additional Supplementary Files

Supplementary Data 1

Reporting Summary

## Data Availability

The publicly available TCGA-BLCA cohort data (the data included 404 tumors, and 19 normal samples) were obtained from the GDC Data Portal website (https://portal.gdc.cancer.gov/). The publicly available GSE data sets (GSE128959, GSE13507, GSE169455, GSE19423, GSE3167, GSE32548, GSE48075, GSE48276, GSE69795, GSE70691, GSE86411, GSE37817) were obtained from the National Center for Biotechnology Information website (https://www.ncbi.nlm.nih.gov/gds/). All data generated or analyzed during this study are included in this article and its Supplementary Information files. The Supplementary Information file contains all Supplementary Figs. (Supplementary Figs. 1-9) and the original uncropped Western blots (Supplementary Fig. 10). The source data behind all graphs in the manuscript are in the Supplementary Data 1.
