# Supplementary material for: Parkin inhibits proliferation and migration of bladder cancer via ubiquitinating Catalase
Source: Commun Biol. 2024 Feb 29;7:245. doi: 10.1038/s42003-024-05935-x (PMC10904755; doi:10.1038/s42003-024-05935-x)
Supplement: Supplementary file 1 — Supplementary Information [file 42003_2024_5935_MOESM1_ESM.pdf]

Supplementary Information

Parkin inhibits proliferation and migration of bladder cancer via ubiquitinating Catalase

Supplementary Figures 1-10: Pages 2-26

Supplementary Tables 1-4: Pages 27-30

Supplementary Figures 1-10

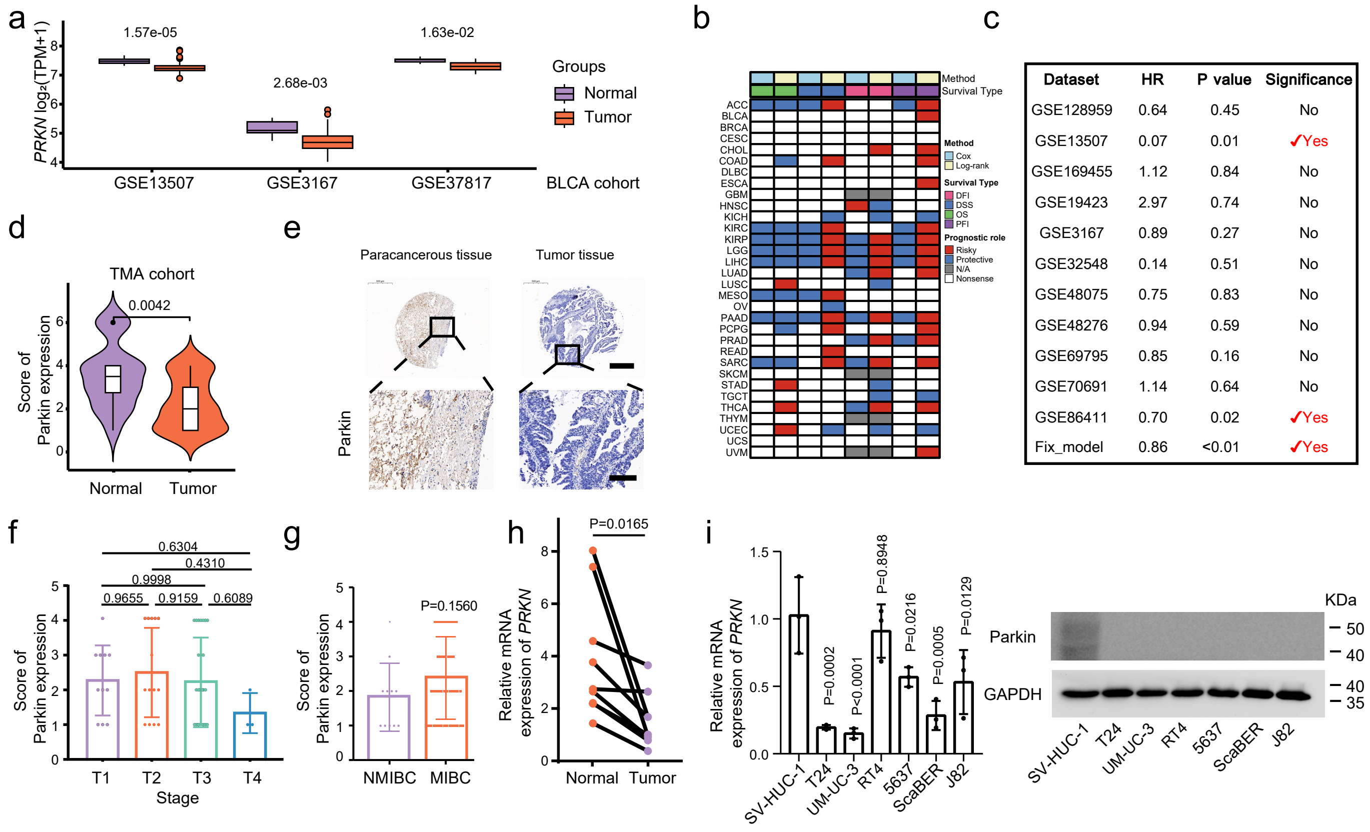

Supplementary Figure 1. Expression and prognostic value of *PRKN* in BLCA.

(a) To compare the expression levels of *PRKN* in BLCA and control samples in the GSE dataset, the Wilcoxon test was used (GSE13507, tumor = 165, normal = 10, p = 1.57e-05; GSE3167, tumor = 51, normal = 9, p = 2.68e-03; GSE37817, tumor = 18, normal = 6, p = 1.63e-02). The data were obtained from the GEO database. **(b)** Heatmap demonstrating the prognostic value of *PRKN* expression in 32 types of cancer in the TCGA cohort examined via univariate Cox regression and survival analysis using four survival cutoff points: overall survival (OS), disease-specific survival (DSS), disease-free interval (DFI), and progression-free interval (PFI). According to the TCGA data, *PRKN* is a risk factor for PFS in patients with BLCA. **(c)** To explore the relationship between *PRKN* expression and BLCA OS based on 11 GEO datasets, we used univariate Cox analysis and showed that *PRKN* expression was a protective factor against BLCA OS in the GSE13507 and GSE86411 cohorts. **(d)** Parkin protein levels were compared between BLCA (n = 63) and normal (n = 16) tissue samples by using the Wilcoxon test (p = 0.0042). **(e)** Representative IHC images of adjacent normal tissue and tumor tissue from the same patient obtained from the TMA are shown. Scale bars:

0.5 mm, 100 μm (enlarged). **(f)** Parkin protein levels were compared among human BLCA samples from different T stages in the TMA (T1 = 11, T2 = 14, T3 = 25, T4 = 3). **(g)** Parkin protein levels were compared between NMIBC and MIBC tissues from the TMA (NMIBC = 11, MIBC = 45). **(h)** The mRNA levels of adjacent normal tissue and BLCA tissues were detected via qRT-PCR in 9 pairs of patients from the Zhongnan Hospital. (n = 9, two-sample paired t-test). **(i)** The mRNA levels of *PRKN* used SV-HUC-1 and six commonly BLCA cell lines were detected using qRT-PCR (n = 3, one-way ANOVA, GAPDH was used as an internal reference). Representative images of the protein expression levels of Parkin in SV-HUC-1 and the six BLCA cell lines were detected using Western blotting and are shown on the right.

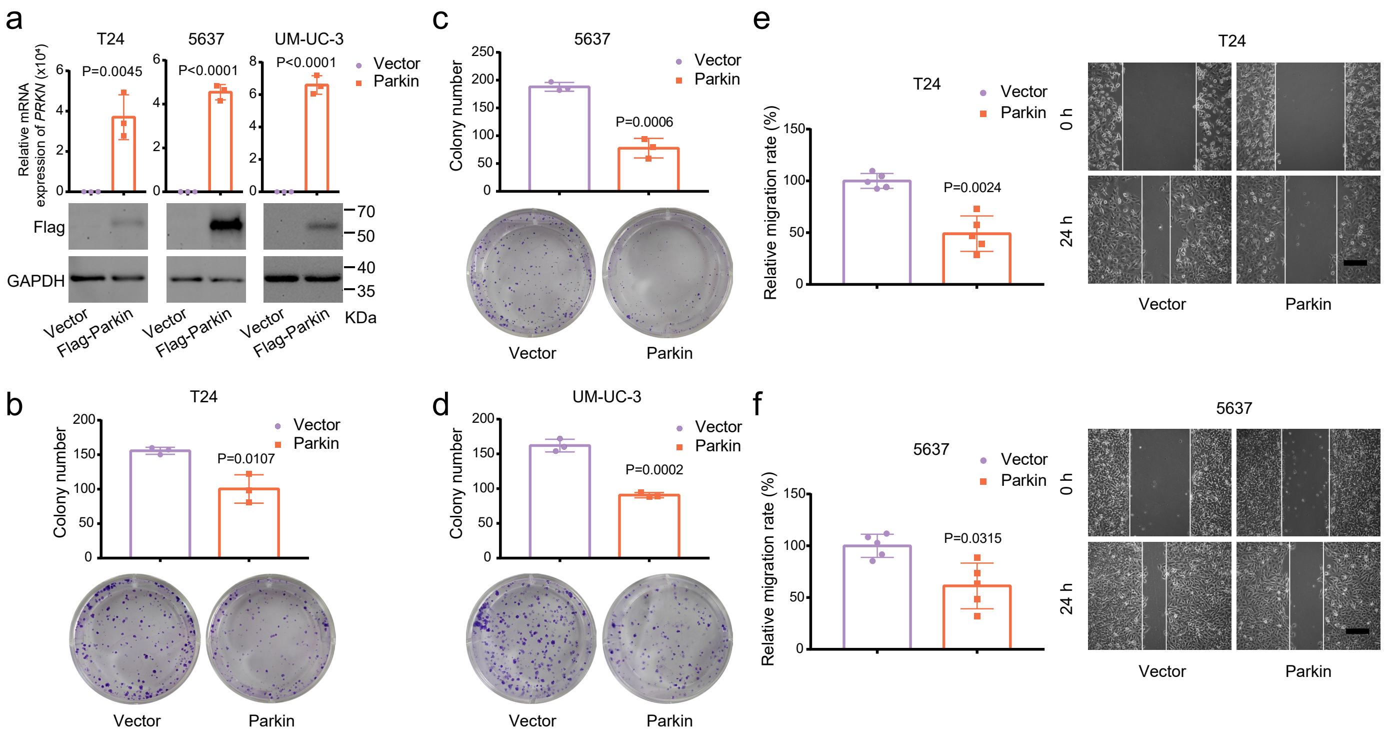

Supplementary Figure 2. Parkin overexpression inhibits BLCA cell proliferation and migration. **(a)** The efficiency of *PRKN* mRNA and protein overexpression in BLCA cells was determined by RT-qPCR and Western blotting, respectively. **(b-d)** A clonogenic assay showed that T24 (b), 5637 (c), and UM-UC-3 (d) cells grew into clusters after overexpressing Parkin (n = 3, unpaired two-tailed Student's t-test). **(e-f)** Wound healing assays showing the migration of T24 (e) and 5637 (f) cells overexpressing Parkin (scale bar: 200 μm, n = 5, unpaired two-tailed Student's t-test). The data are presented as the means ± SD. Exact *p* values are shown. The n number represents n biologically independent experiments in each group.

Supplementary Figure 3

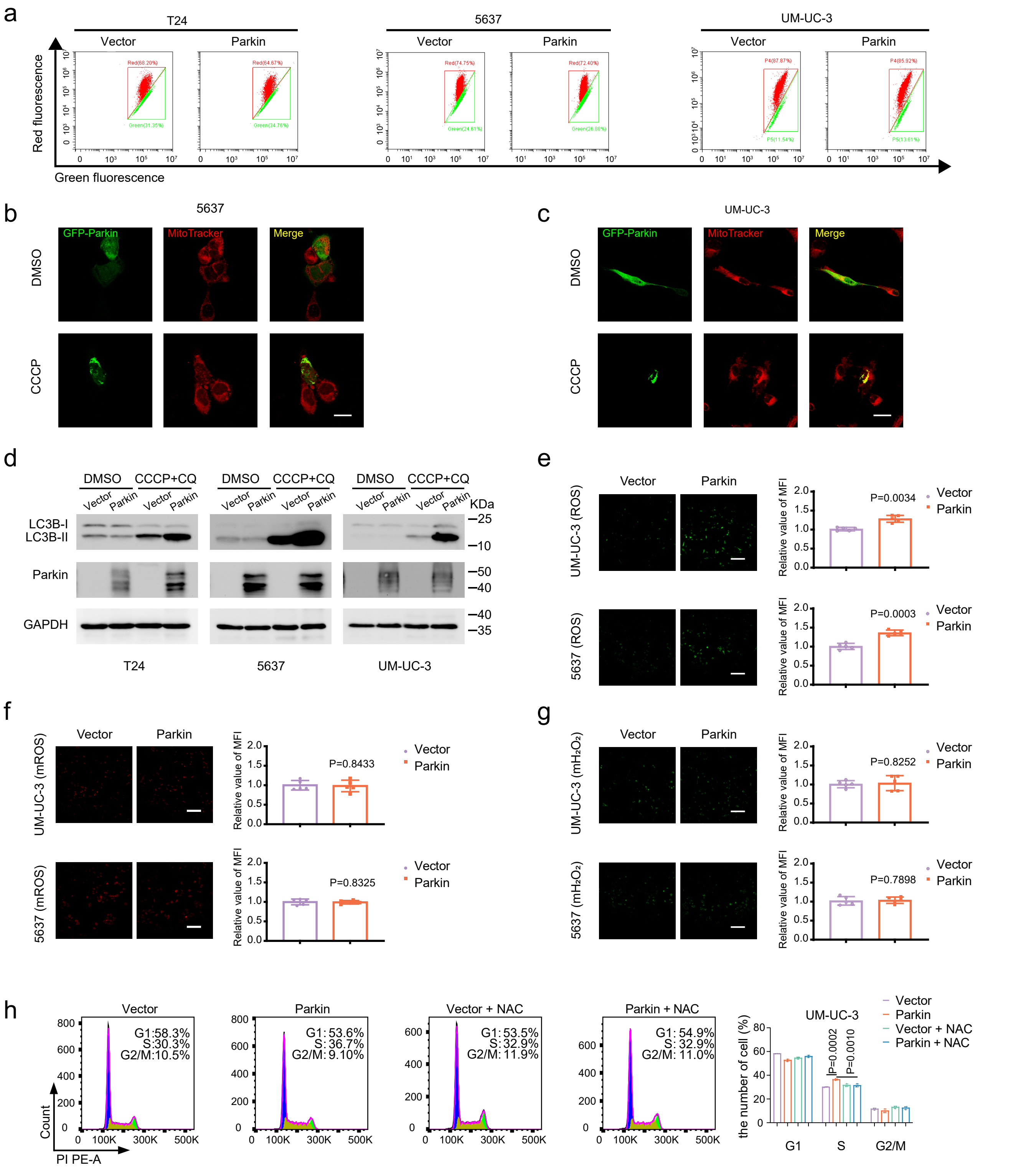

Supplementary Figure 3. Parkin upregulation increased intracellular ROS levels in an autophagy-independent manner.

(a) The mitochondrial membrane potential was detected by the JC-1 kit and flow cytometry in T24, 5637,

Supplementary Figure 3

---

and UM-UC-3 cells. **(b-c)** Representative immunofluorescence images of GFP-Parkin-overexpressing 5637 (B) and UM-UC-3 (C) cells labeled with control and CCCP-treated mitochondria are shown. Scale bar: 20 μm. **(d)** Changes in the protein levels of LC3B-I and LC3B-II were examined in stable Parkin-overexpressing T24, 5637, and UM-UC-3 cells following treatment with DMSO or CCCP + CQ. **(e)** The intracellular ROS levels were measured by DCFH-DA via immunofluorescence in UM-UC-3 and 5637 cells (scale bar: 120 μm, n = 5, unpaired two-tailed Student's t-test). **(f-g)** The mitochondrial ROS levels and mitochondrial H_2O_2 levels were measured by immunofluorescence staining for MitoSOX Red (f) and MitoPY1 (g) in UM-UC-3 and 5637 cells (scale bar: 120 μm, n = 5, unpaired two-tailed Student's t-test). **(h)** Cell cycle analysis of UM-UC-3 cells transfected with empty vector or Parkin was performed by flow cytometry with or without the addition of NAC (n = 3). One-way ANOVA was used to analyze the means ± SD. Exact *p* values are shown. The n number represents n biologically independent experiments in each group.

Supplementary Figure 4

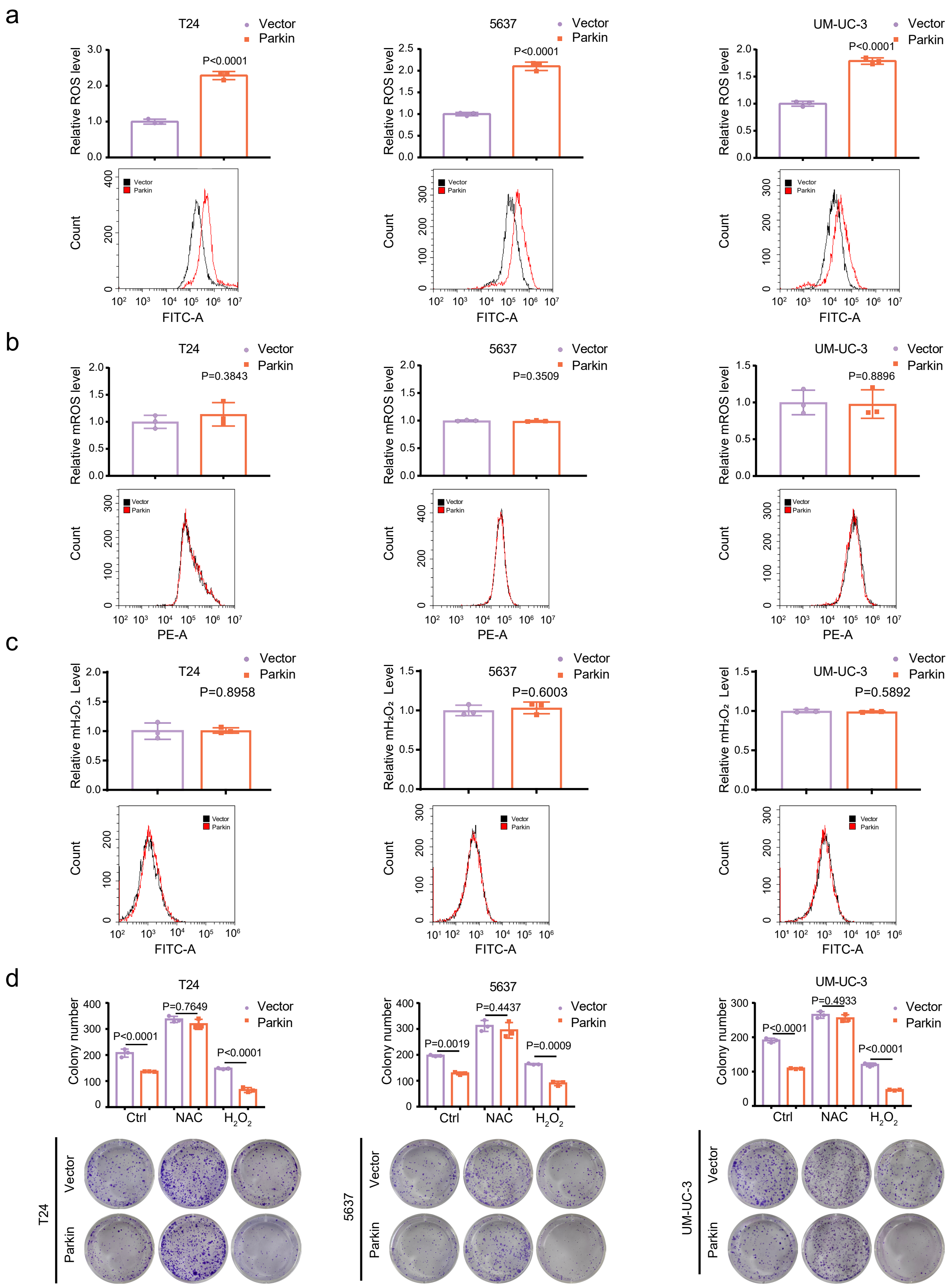

Supplementary Figure 4. Parkin upregulation increased intracellular ROS levels.

Supplementary Figure 4

(a) The intracellular ROS levels in T24, 5637, and UM-UC-3 cells were measured via flow cytometry with DCFH-DA (n = 3, unpaired two-tailed Student's t-test). **(b)** The mitochondrial ROS levels in T24, 5637, and UM-UC-3 cells were measured via MitoSOX Red via flow cytometry (n = 3, unpaired two-tailed Student's t-test). **(c)** The mitochondrial H_2O_2 levels in T24, 5637, and UM-UC-3 cells were measured via flow cytometry (n = 3, unpaired two-tailed Student's t-test). **(d)** A clonogenic assay showed that Parkin-overexpressing T24, 5637, and UM-UC-3 cells grew into clusters after treatment with DMSO, NAC, or H_2O_2. (n = 3, one-way ANOVA). The data are presented as the means ± SD. Exact *p* values are shown. The n number represents n biologically independent experiments in each group.

Supplementary Figure 5

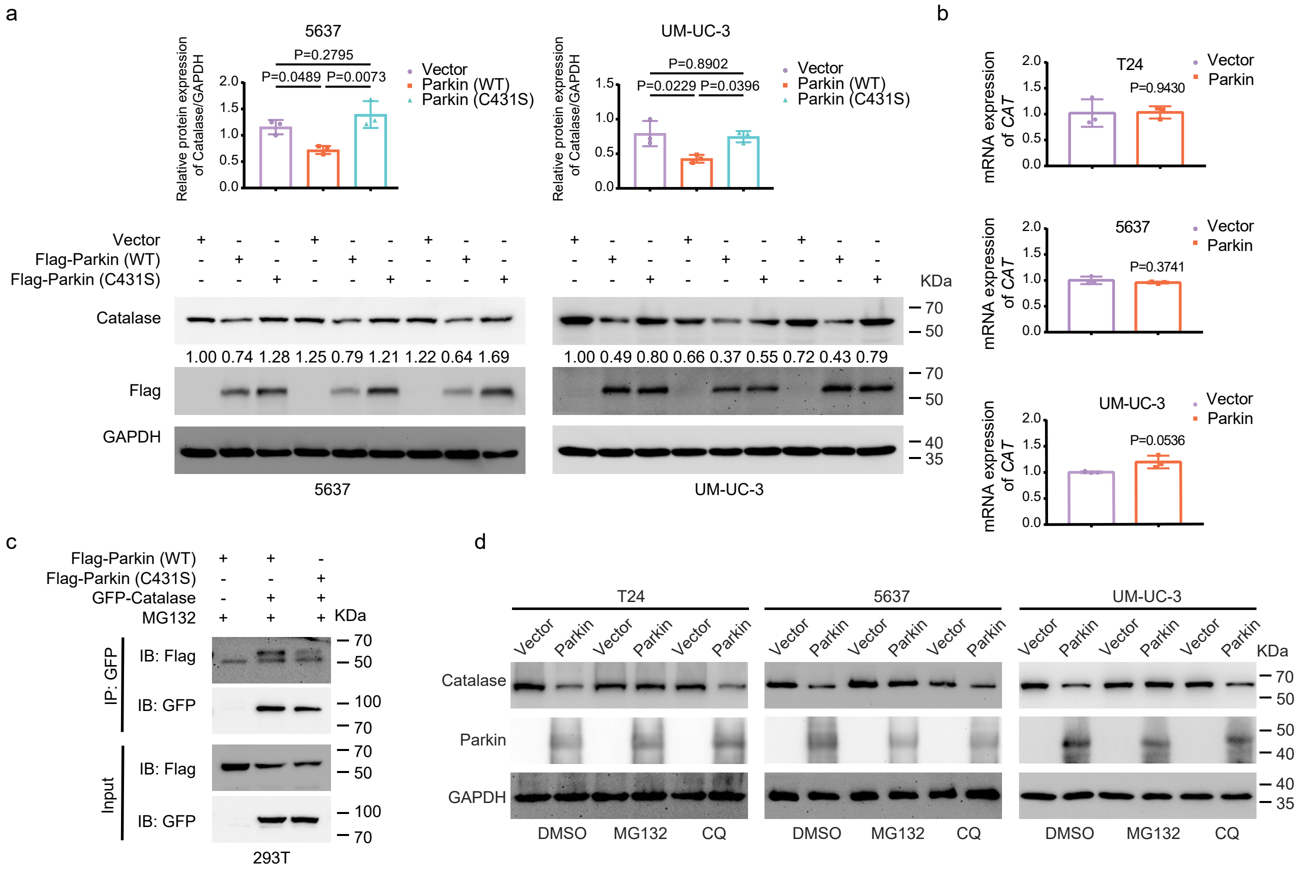

Supplementary Figure 5. Parkin interacts with Catalase and decreases its protein expression level. **(a)** Western blot analysis revealed alterations in Catalase protein levels following the overexpression of Parkin and Parkin (C431S) in 5637 and UM-UC-3 cells (n = 3, one-way ANOVA). **(b)** The relative mRNA level of *CAT* in T24, 5637, and UM-UC-3 cells after Parkin was upregulated determined via qRT-PCR (n = 3, unpaired two-tailed Student's t-test). **(c)** The presence of exogenous Parkin, Parkin (C431S), and Catalase was detected in 293T cells using a co-IP assay. **(d)** Changes in the protein levels of Catalase were examined in stable Parkin-overexpressing T24, 5637, and UM-UC-3 cells following treatment with DMSO, MG132 or CQ. The data are presented as the means ± SD. Exact *p* values are shown. The n number represents n biologically independent experiments in each group.

Supplementary Figure 6

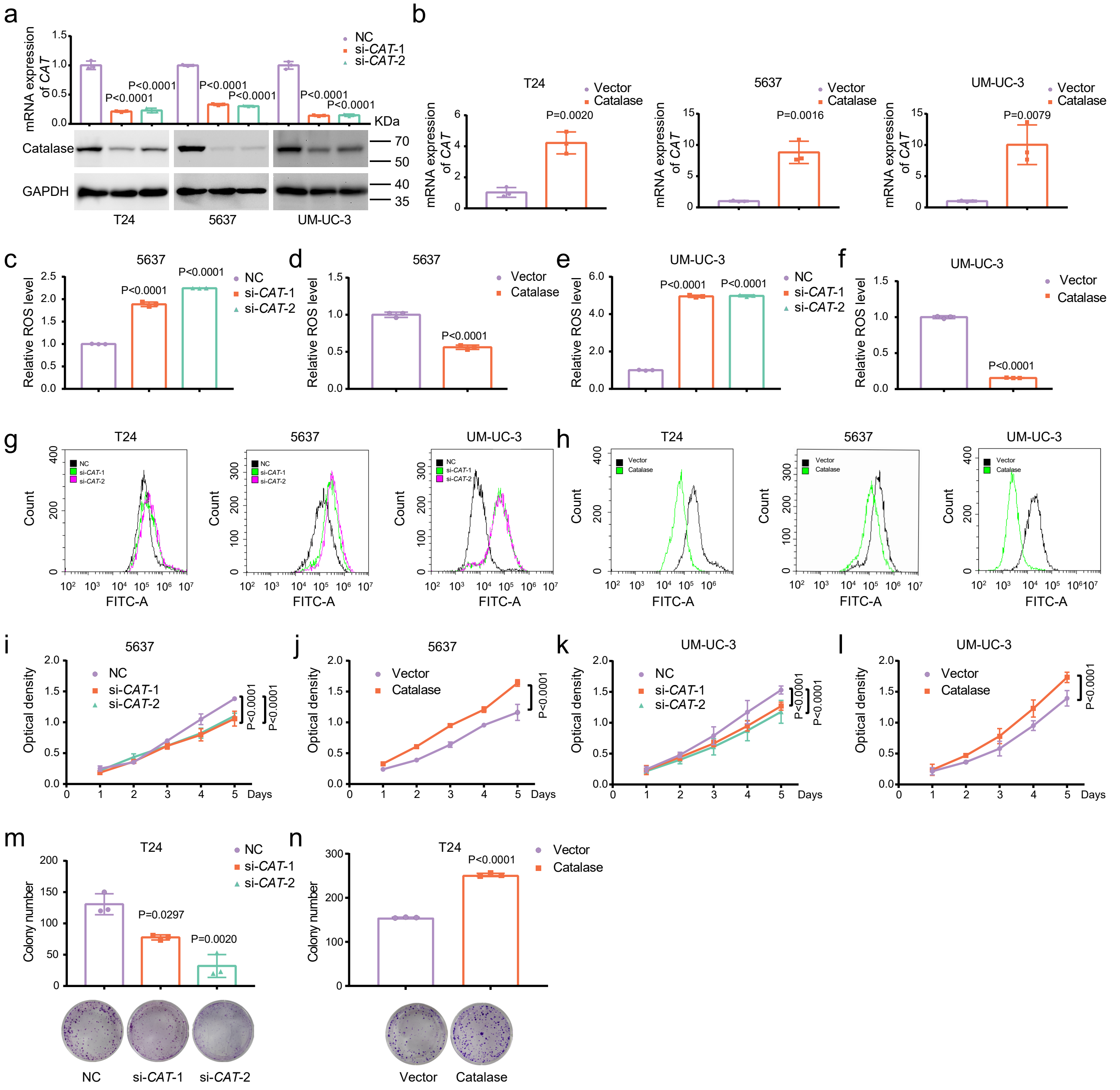

Supplementary Figure 6. Catalase overexpression promotes cell proliferation and migration, and *CAT* knockdown has the opposite effect.

(a) The efficiency of knocking down *CAT* mRNA and protein expression in BLCA cells was determined by RT-qPCR (n = 3, one-way ANOVA) and Western blot, respectively. **(b)** The efficiency of overexpressing *CAT* mRNA in BLCA cells was determined via RT-qPCR (n = 3, unpaired two-tailed Student's t-test). **(c-f)** The intracellular ROS levels were measured after *CAT* knockdown in 5637 (c) and UM-UC-3 (e) cells (n = 3, one-way ANOVA) and after Catalase overexpression in 5637 (d) and UM-UC-3 (f) cells (n = 3, unpaired

two-tailed Student's t-test) by DCFH-DA via flow cytometry. **(g-h)** The intracellular ROS levels were measured after *CAT* knockdown (g) and Catalase overexpression (h) by DCFH-DA via flow cytometry in BLCA cells. **(i-l)** The MTT assay indicates the proliferative capacity after knockdown of *CAT* in 5637 (i) and UM-UC-3 (k) cells and overexpression of Catalase in 5637 (j) and UM-UC-3 (l) cells (n = 8, two-way ANOVA). **(m-n)** A clonogenic assay was used to evaluate cell viability after knockdown of *CAT* in T24 (m) cells (n = 3, one-way ANOVA) or after overexpression of Catalase in T24 (n) cells (n = 3, unpaired two-tailed Student's t-test). The data are presented as the means ± SD. Exact *p* values are shown. The n number represents n biologically independent experiments in each group.

Supplementary Figure 7

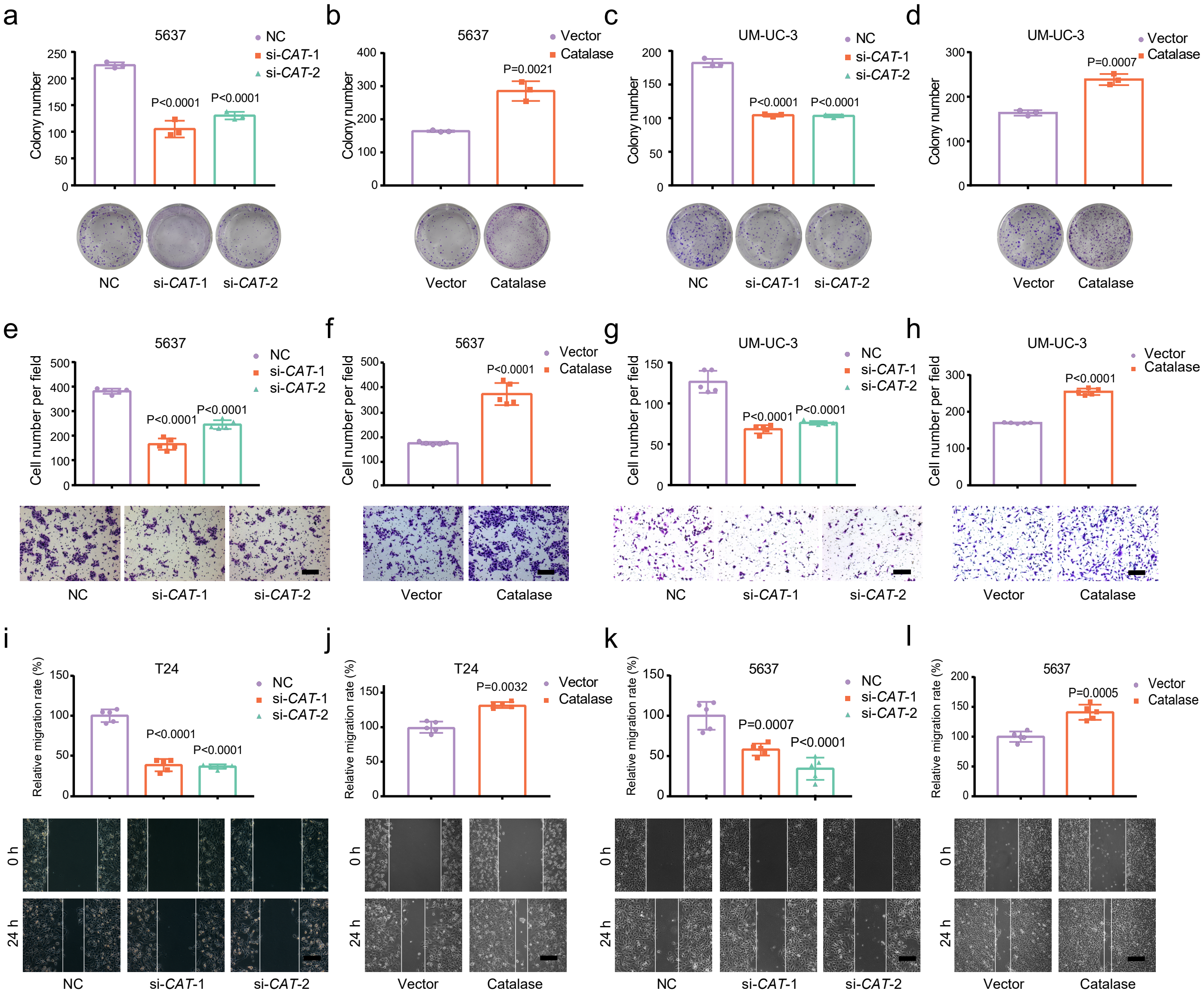

Supplementary Figure 7. Knockdown of *CAT* attenuates cell proliferation and migration, and Catalase upregulation has the opposite effect.

(a-d) A clonogenic assay was used to evaluate cell viability after knockdown of *CAT* in 5637 (a) and UM-UC-3 (c) cells (n = 3, one-way ANOVA) and after overexpression of Catalase in 5637 (b) and UM-UC-3 (d) cells (n = 3, unpaired two-tailed Student's t-test). **(e-h)** Transwell assays showing the migration capacity of 5637 (e) and UM-UC-3 (g) cells after *CAT* knockdown and Catalase-overexpressing 5637 (f) and UM-UC-3 (h) cells (scale bar: 200 μm, n = 5, one-way ANOVA (e, g); unpaired two-tailed Student's t-test (f, h)). **(i-l)** A wound healing assay indicated mobility after *CAT* knockdown in T24 (i) and 5637 (k) cells and Catalase overexpression in T24 (j) and 5637 (l) cells (scale bar: 200 μm, n = 5, one-way ANOVA (i, k), unpaired two-tailed Student's t-test (j, l)). The data are presented as the means ± SD. Exact *p* values are shown. The n number represents n biologically independent experiments in each group.

Supplementary Figure 8

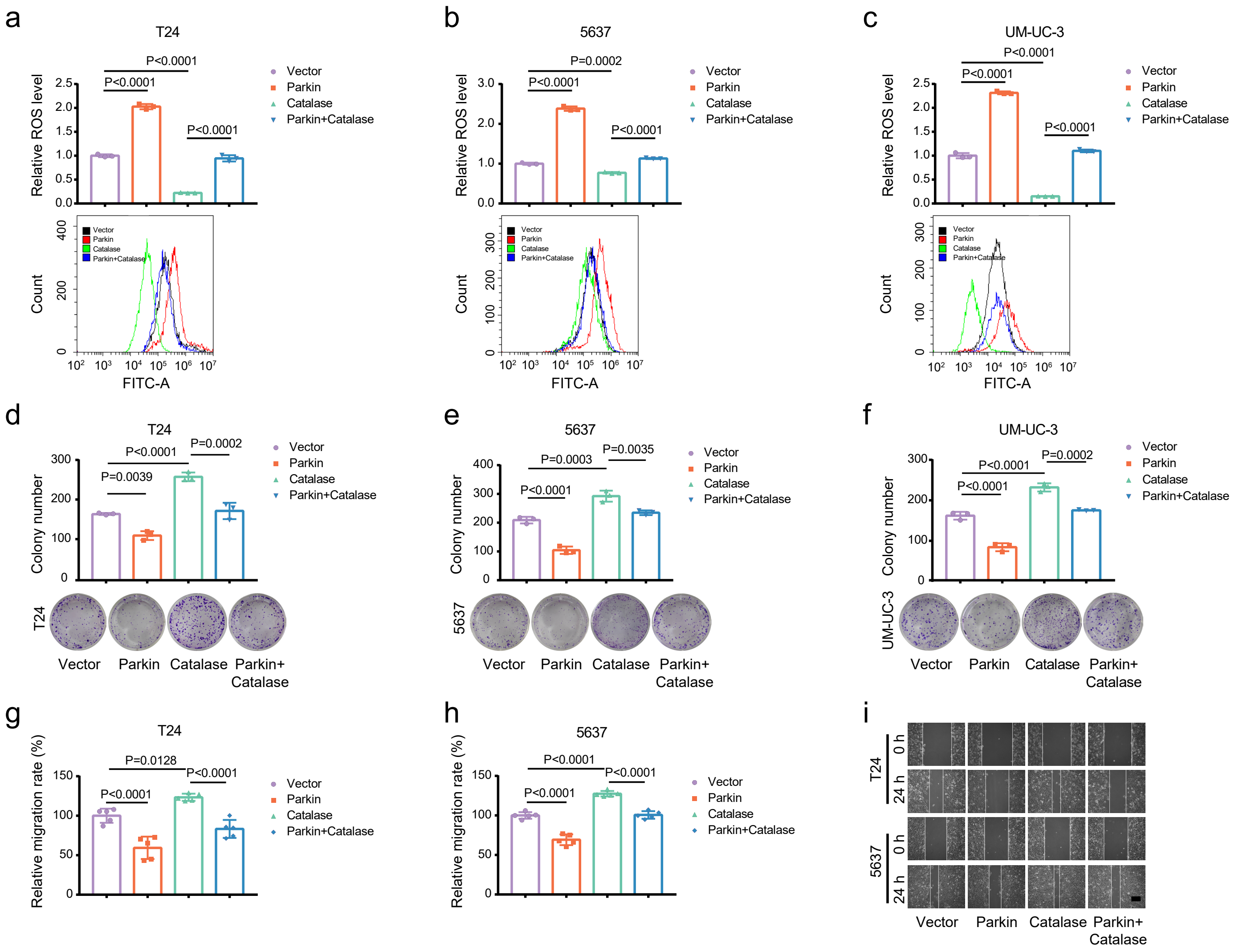

Supplementary Figure 8. Parkin suppressed proliferation and migration via Catalase.

(a-c) Intracellular ROS levels were detected after T24 (a), 5637 (b), and UM-UC-3 (c) cells were overexpressing the empty vector, Parkin, Catalase or Parkin + Catalase (n = 3, one-way ANOVA). **(d-f)** A clonogenic assay was used to evaluate cell viability after the overexpression of the empty vector, Parkin, Catalase or Parkin + Catalase in T24 (d), 5637 (e), and UM-UC-3 (f) cells (n = 3, one-way ANOVA). **(g-i)** A wound healing assay was used to assess the mobility of T24 (g) and 5637 (h) cells after the overexpression of the empty vector, Parkin, Catalase or Parkin + Catalase (n = 5, one-way ANOVA). The data are presented as the means ± SD. Exact *p* values are shown. The n number represents n biologically independent experiments in each group.

Supplementary Figure 9. Gating strategy for flow cytometry.

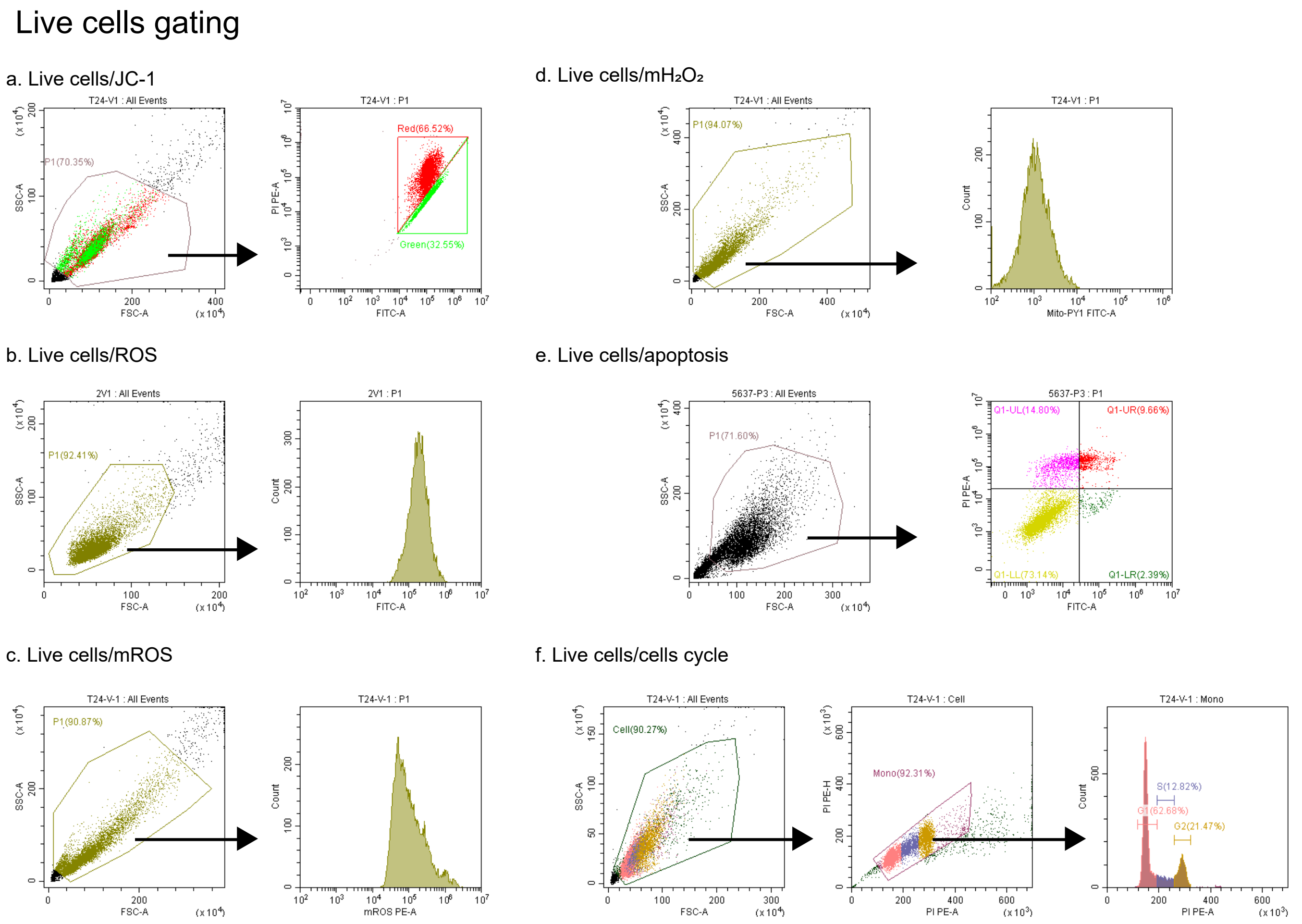

Supplementary Figure 9. Gating strategy for flow cytometry.

JC-1-stained **(a)**, DCFH-DA-stained **(b)**, MitoSOX Red-stained **(c)**, MitoPY1-stained **(d)**, annexin V and PI-stained **(e)**, and PI-stained **(f)** in BLCA cells. Live cells were selected, followed by single cells and then the corresponding laser channels were used to identify. The data are shown for Fig. 3k-l, Fig. 5a-b, Supplementary Fig. 3a and h, Supplementary Fig. 4a-c, Supplementary Fig. 6c-h, and Supplementary Fig. 8a-c. The same gating strategy was used for all above.

Fig. 2g

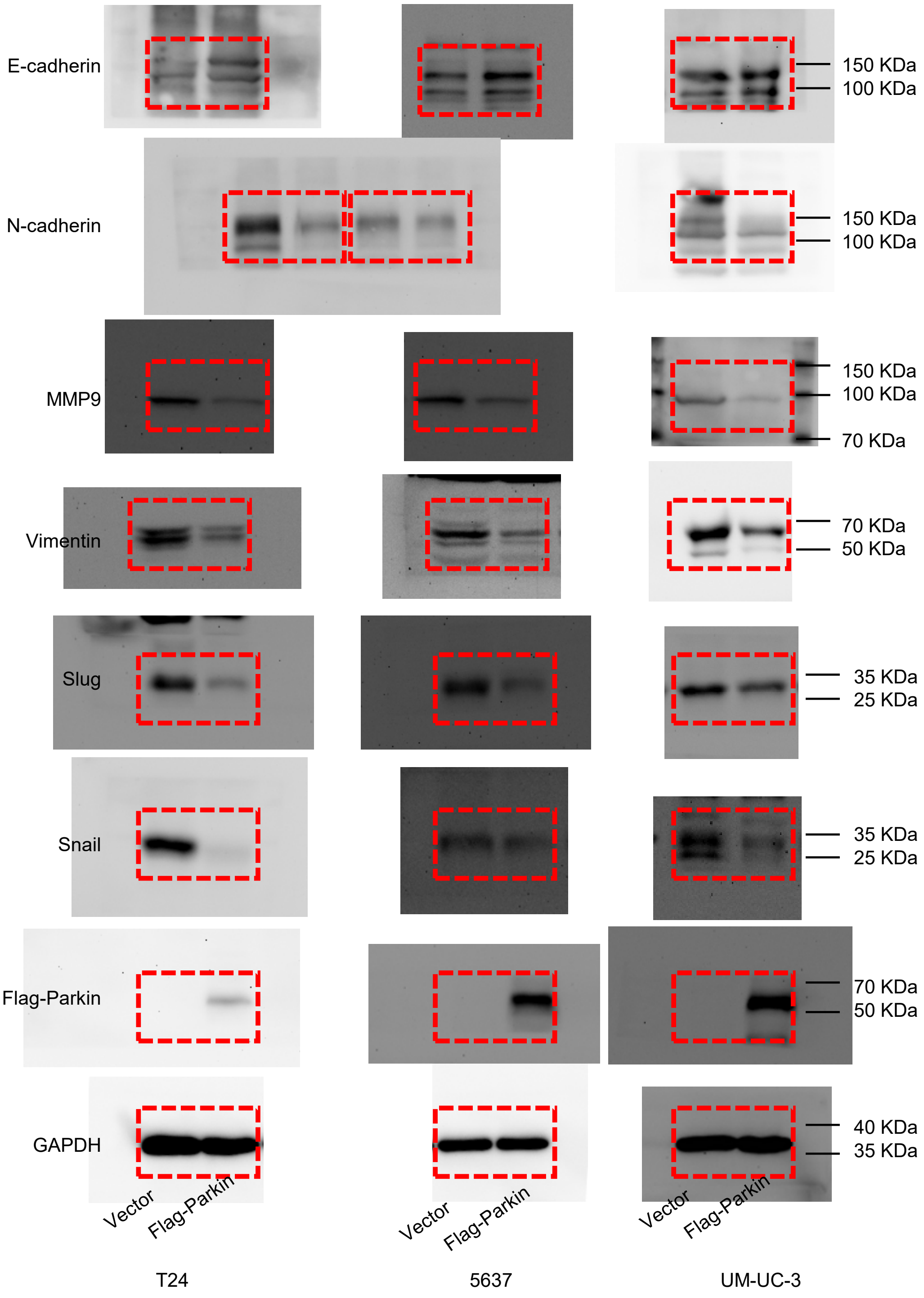

Supplementary Figure 10. Original uncropped Western blots.

Before probing the blots with the indicated antibodies, the membranes were cut to facilitate parallel processing of distinct antigens with their respective antibodies.

Supplementary Figure 10. Original uncropped Western blots.

Fig. 3b

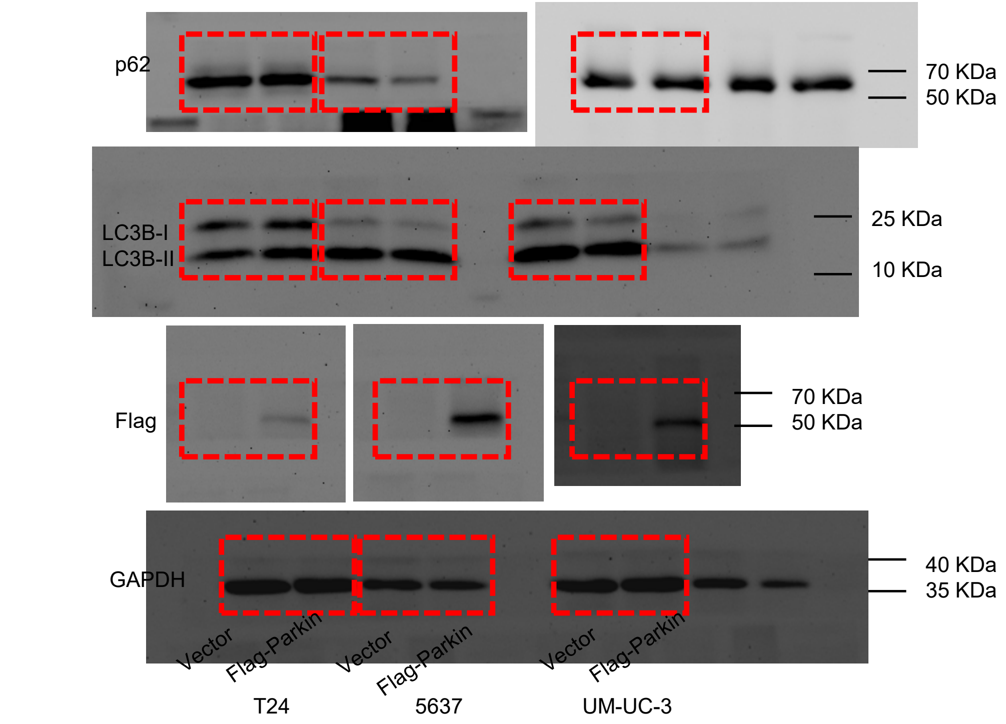

Fig. 4b

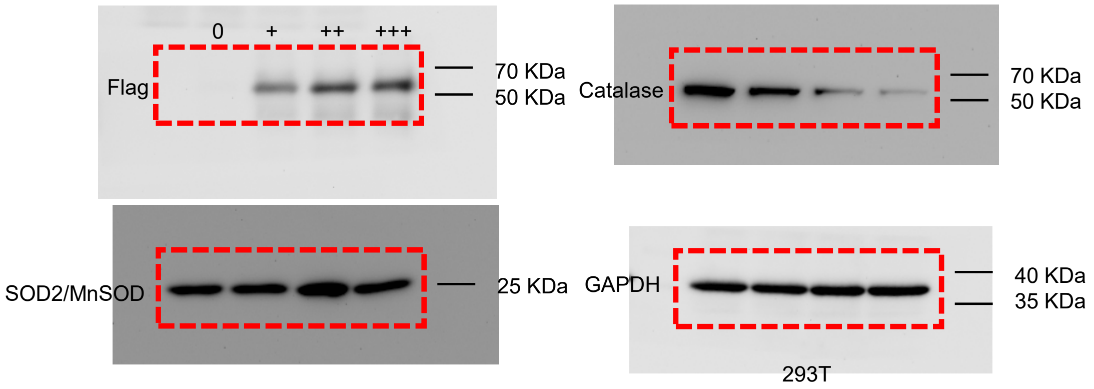

Fig. 4c

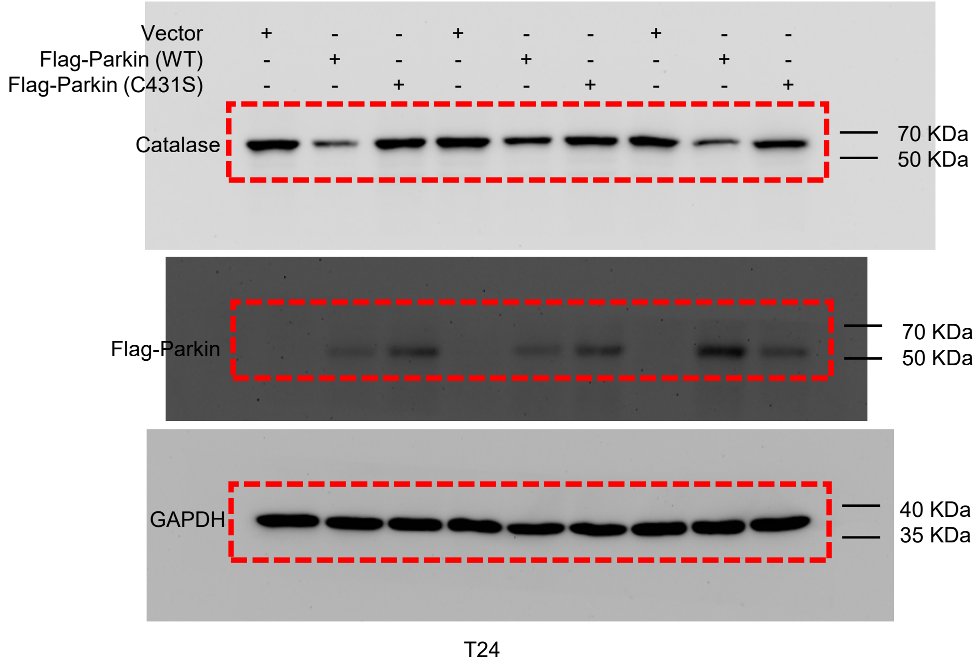

Supplementary Figure 10. Original uncropped Western blots.

Fig. 4d

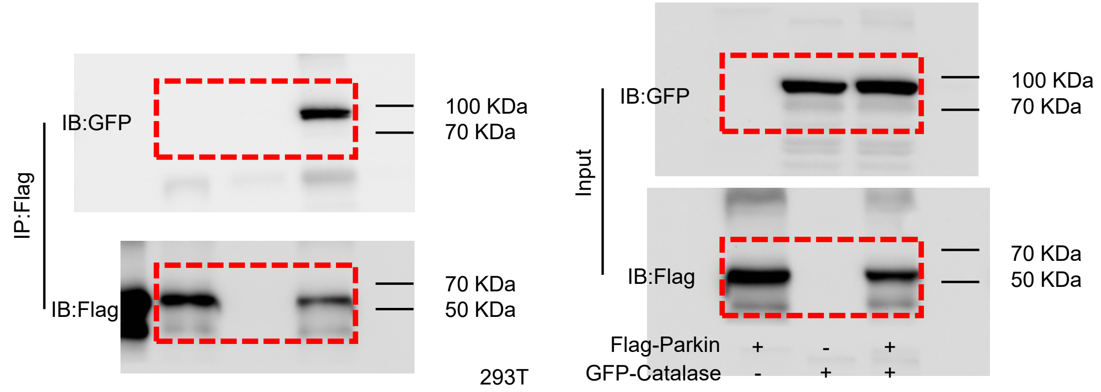

Fig. 4e

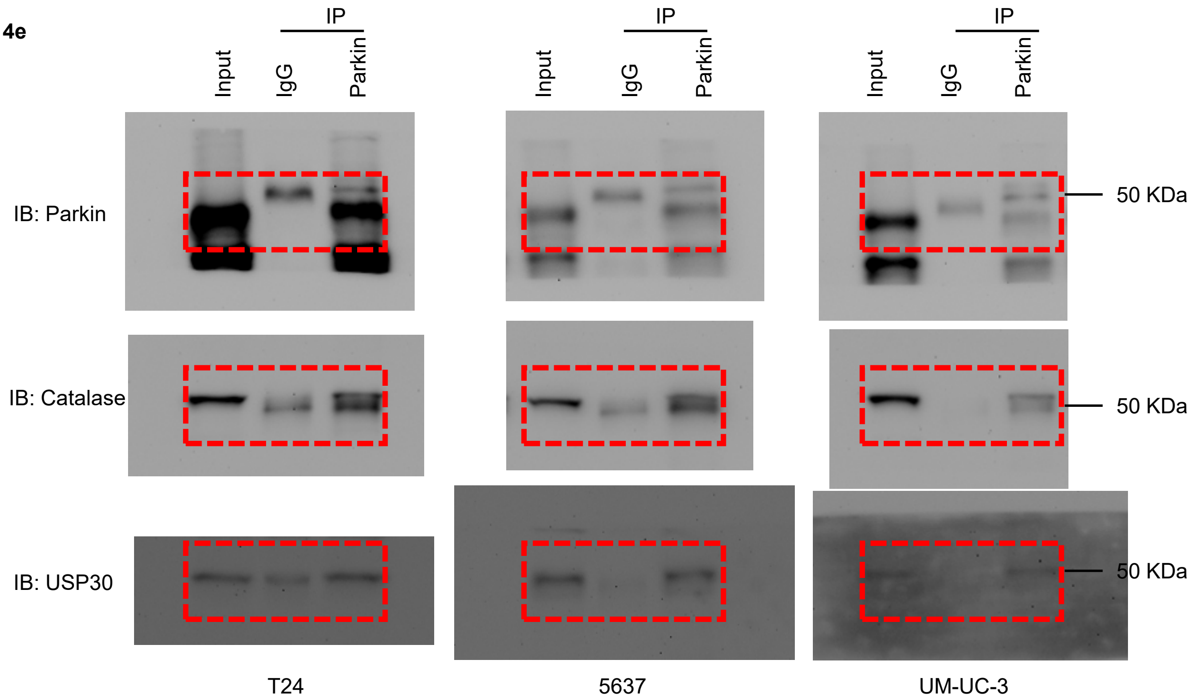

Fig. 4f

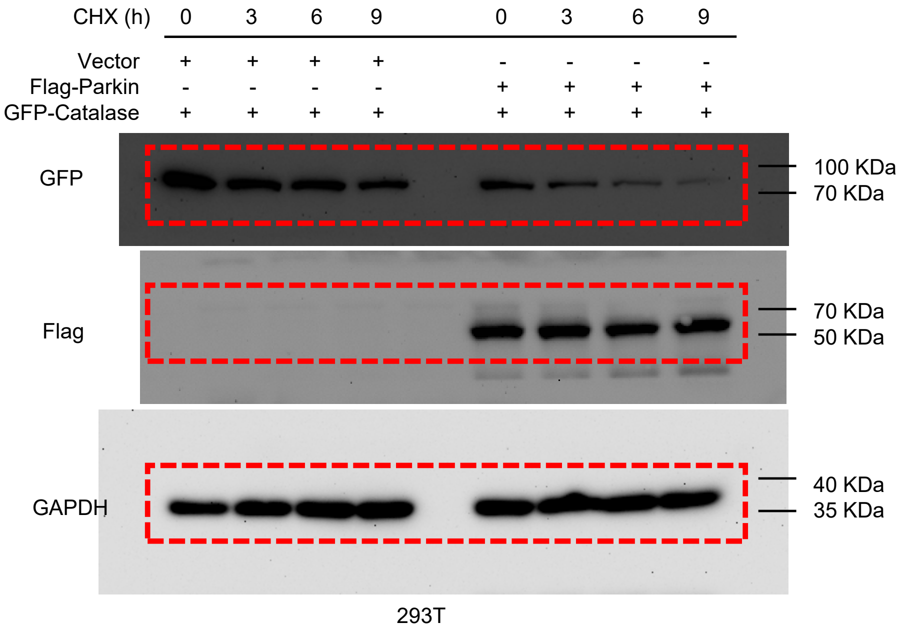

Supplementary Figure 10. Original uncropped Western blots.

Fig. 4g

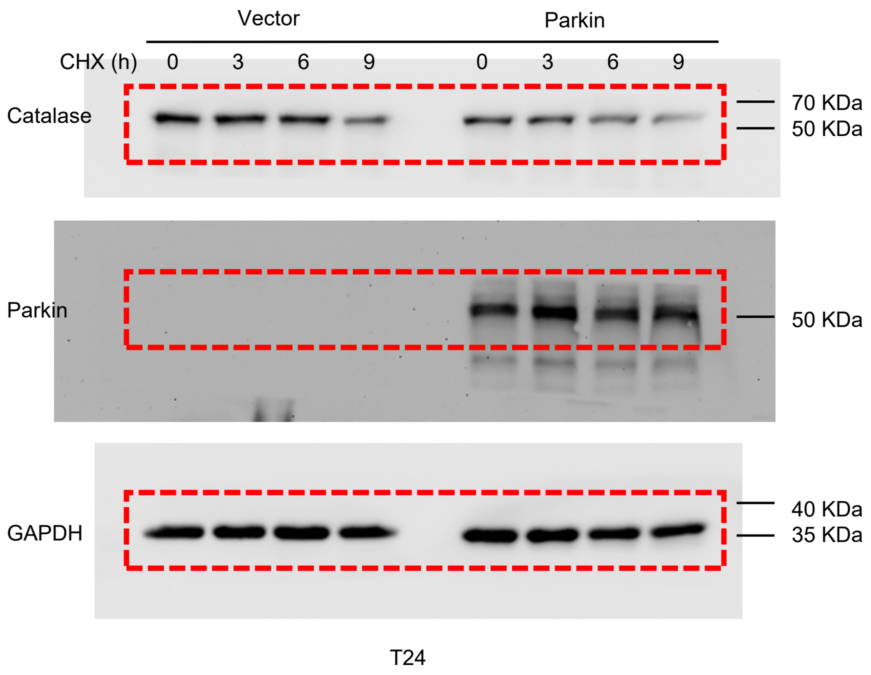

Fig. 4h

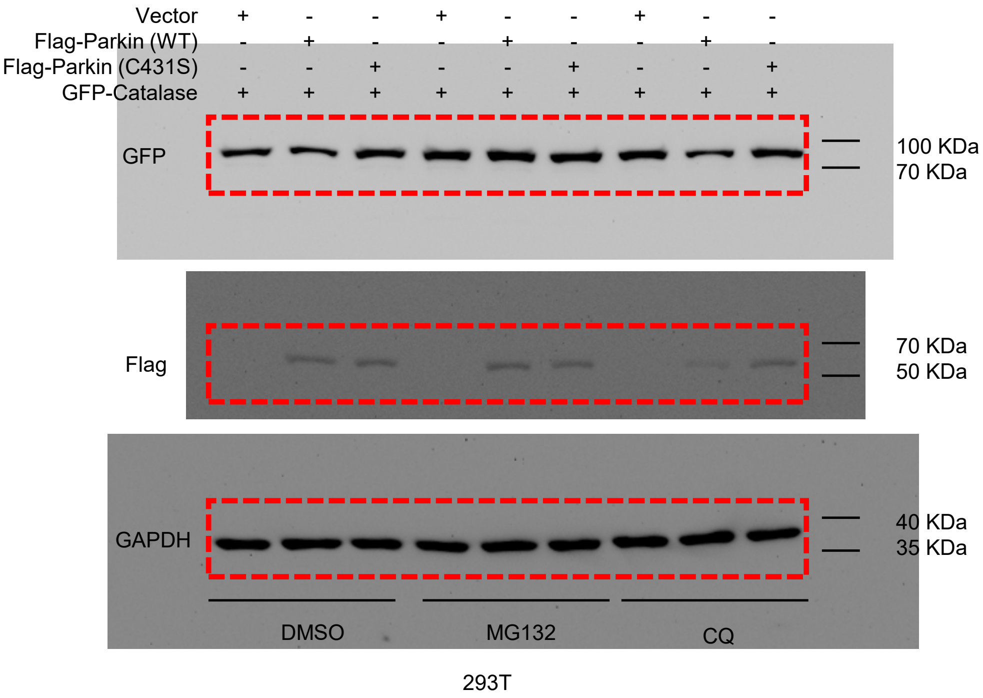

Supplementary Figure 10. Original uncropped Western blots.

Fig. 4i

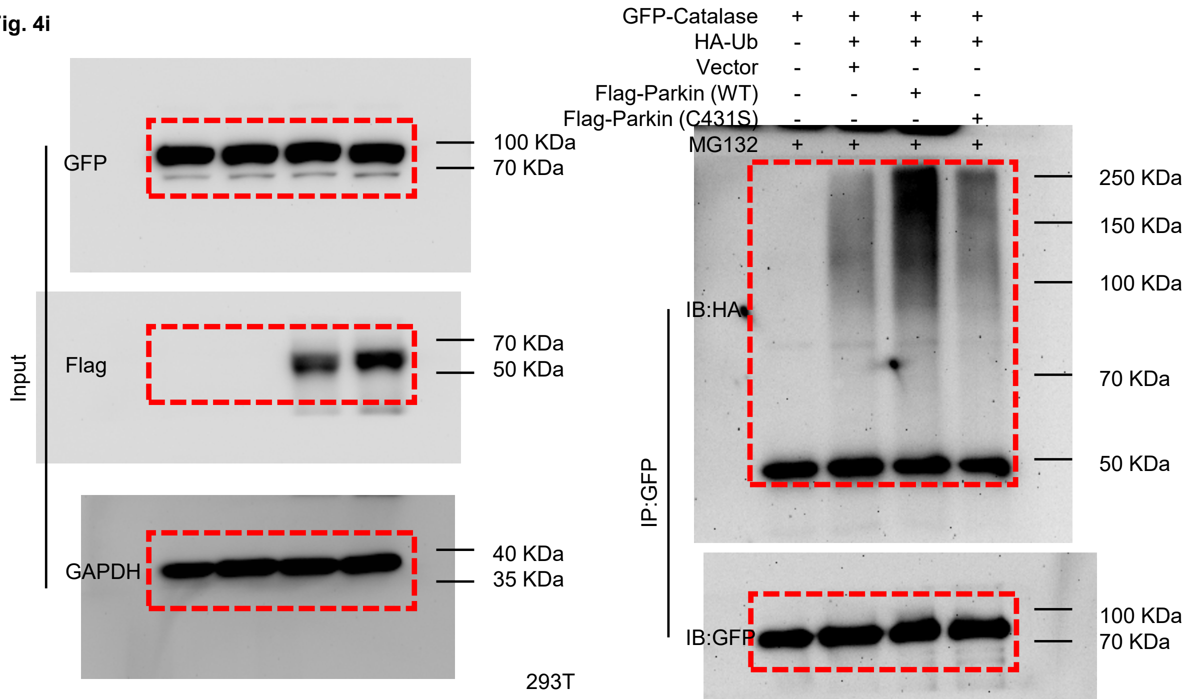

Fig. 5g

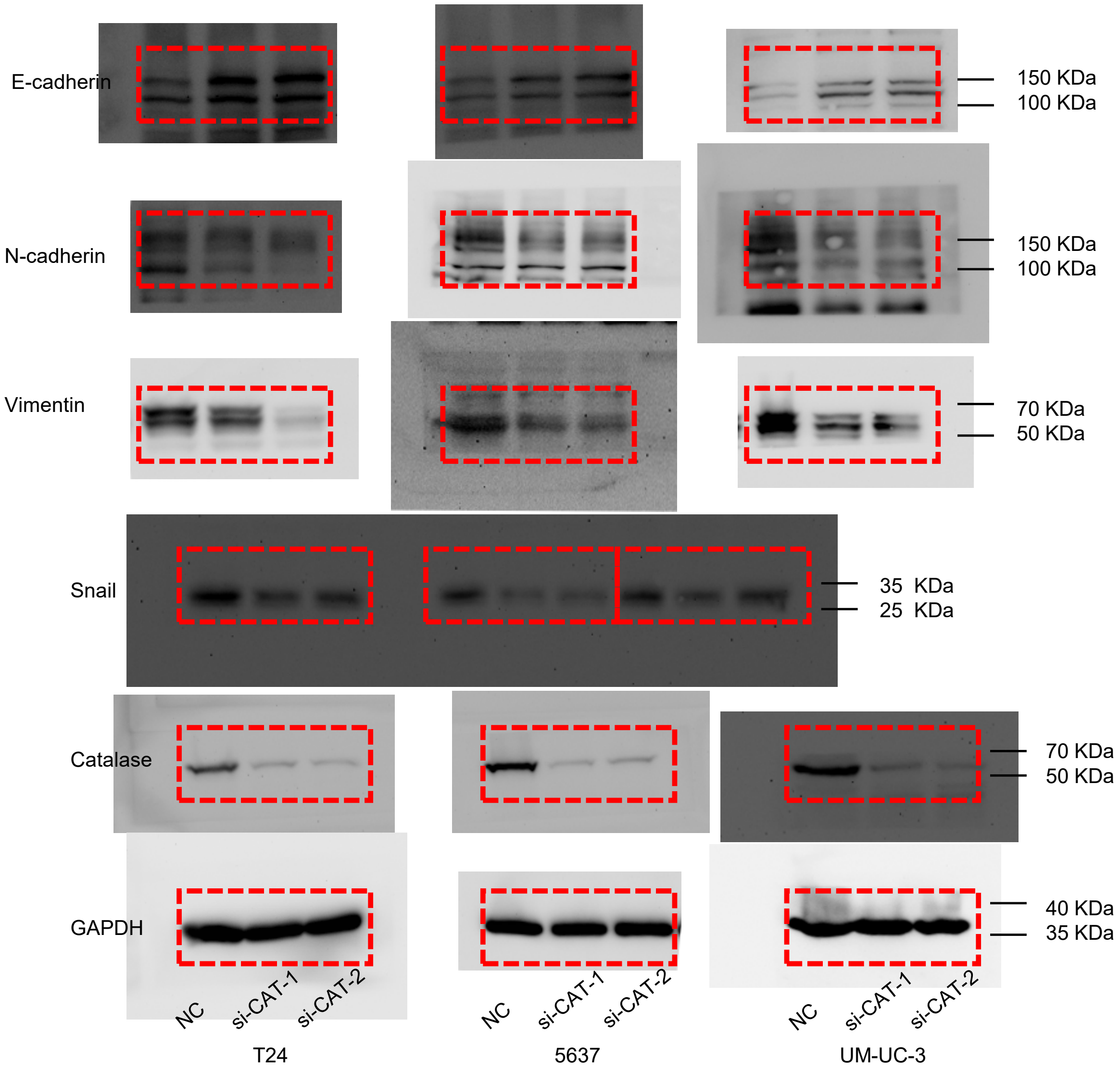

Supplementary Figure 10. Original uncropped Western blots.

Fig. 5h

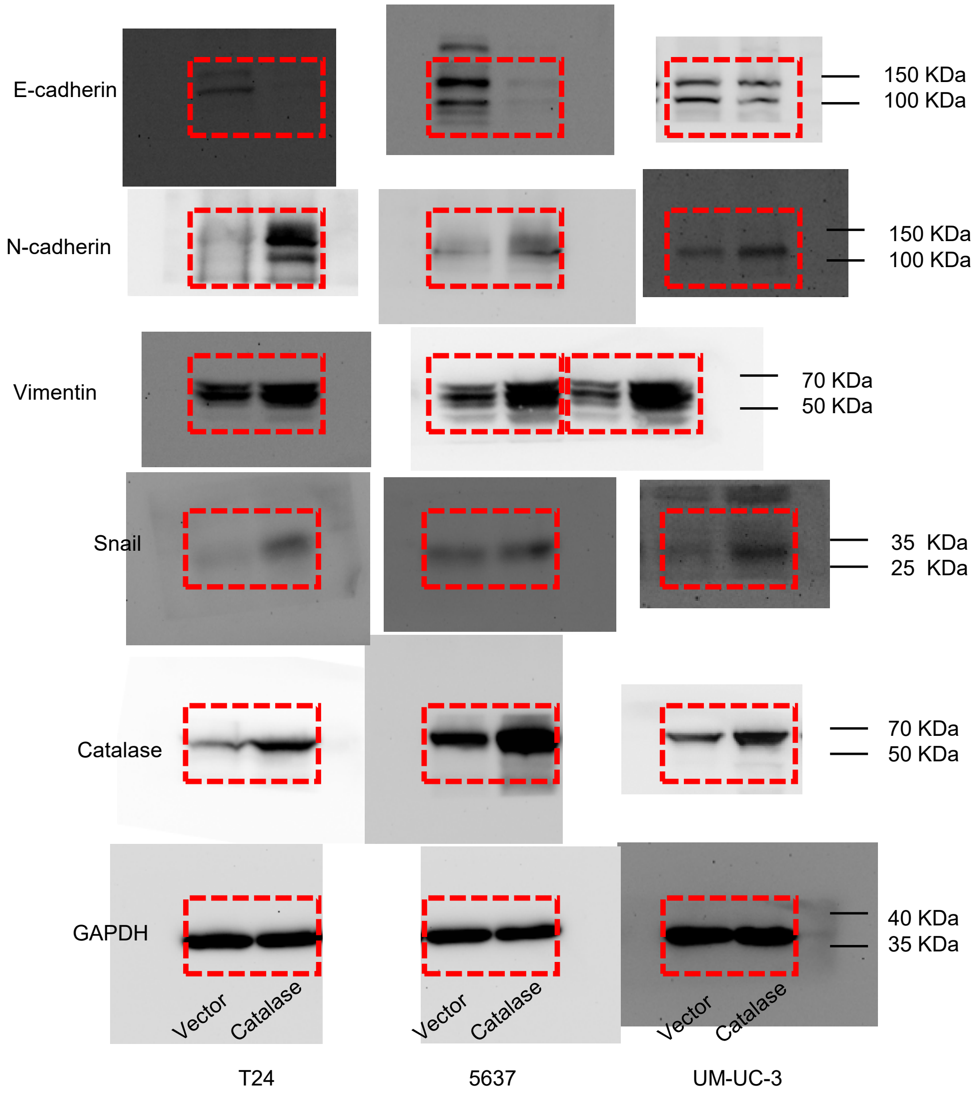

Fig. 6a

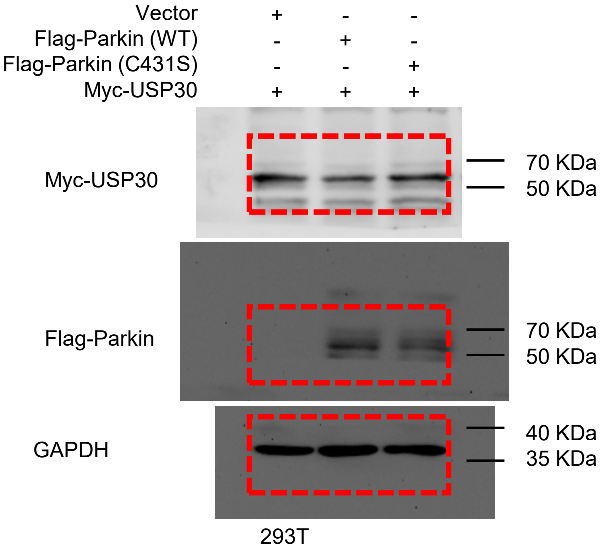

Supplementary Figure 10. Original uncropped Western blots.

Fig. 6b

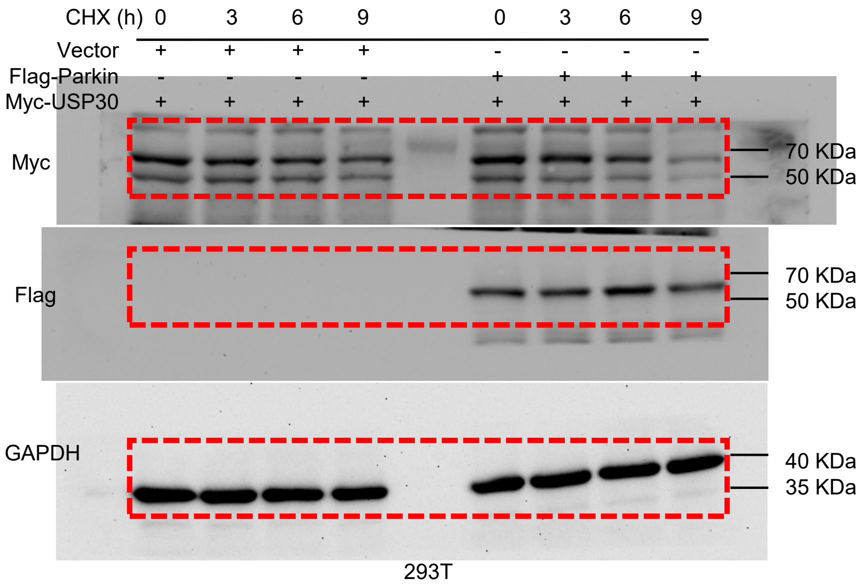

Fig. 6c

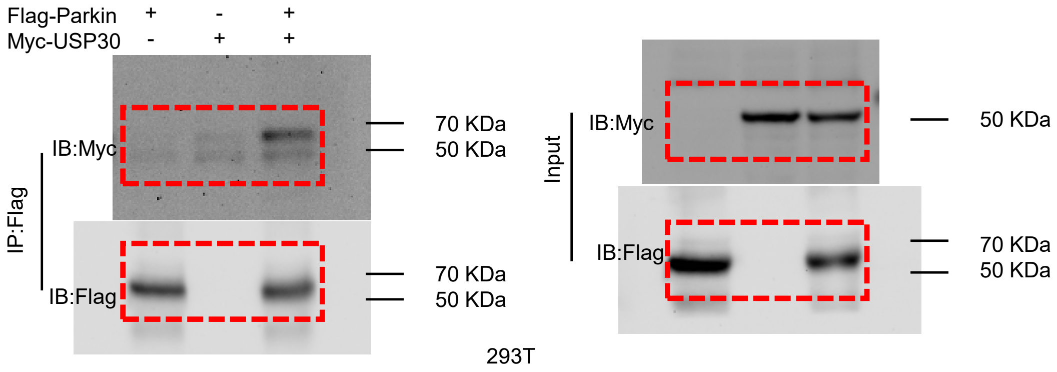

Fig. 6e

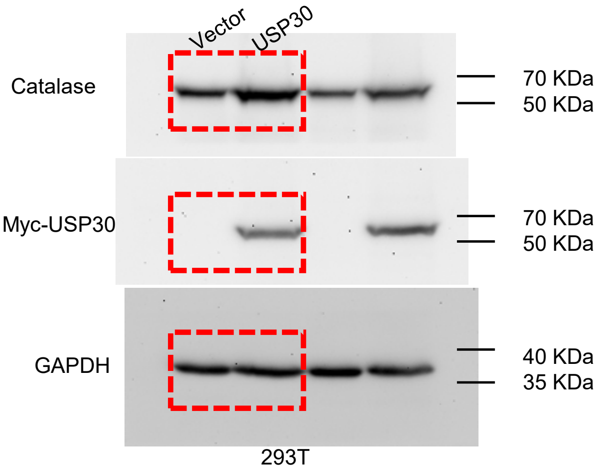

Fig. 6f

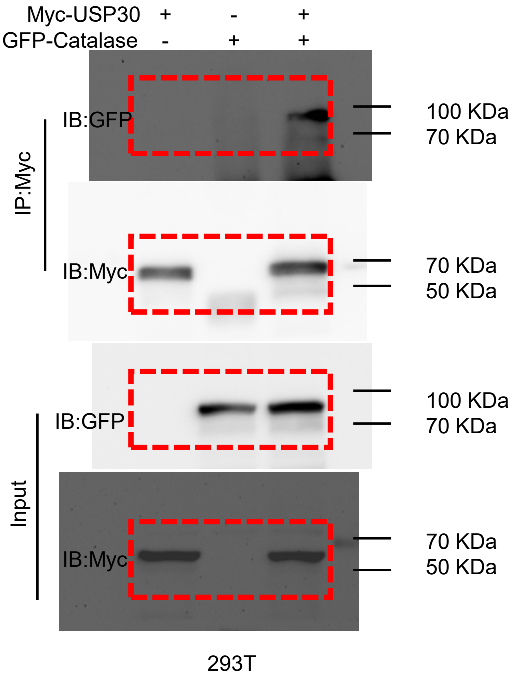

Supplementary Figure 10. Original uncropped Western blots.

Fig. 6g

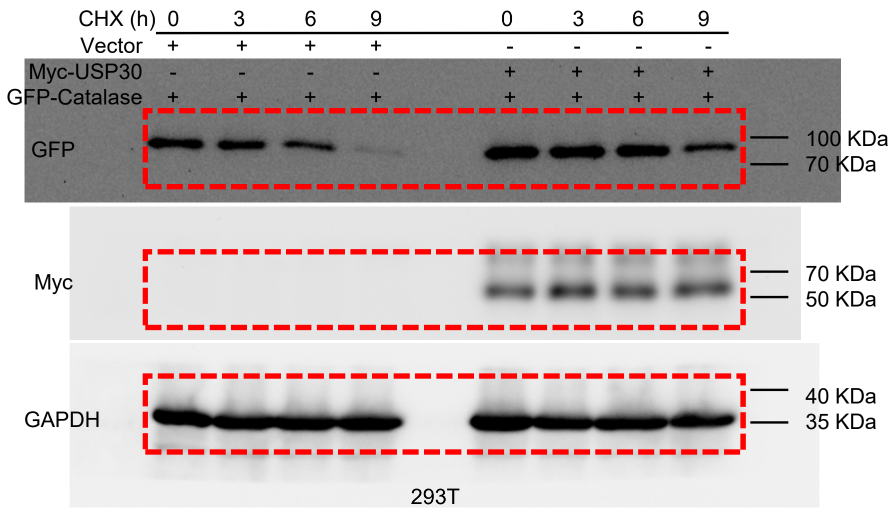

Fig. 6h

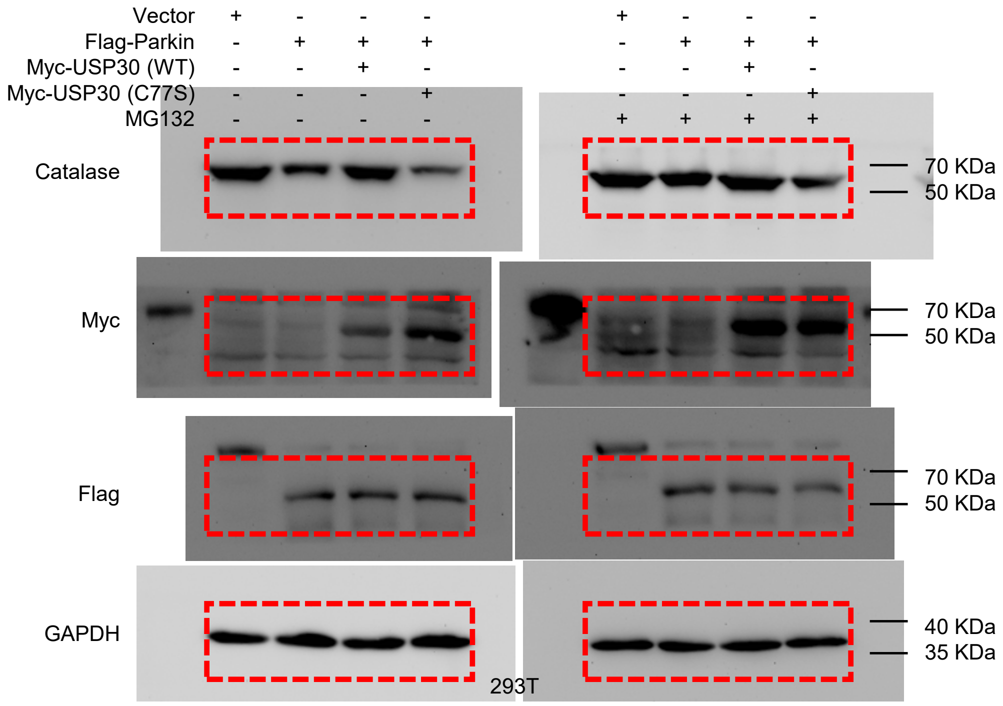

Fig. 6i

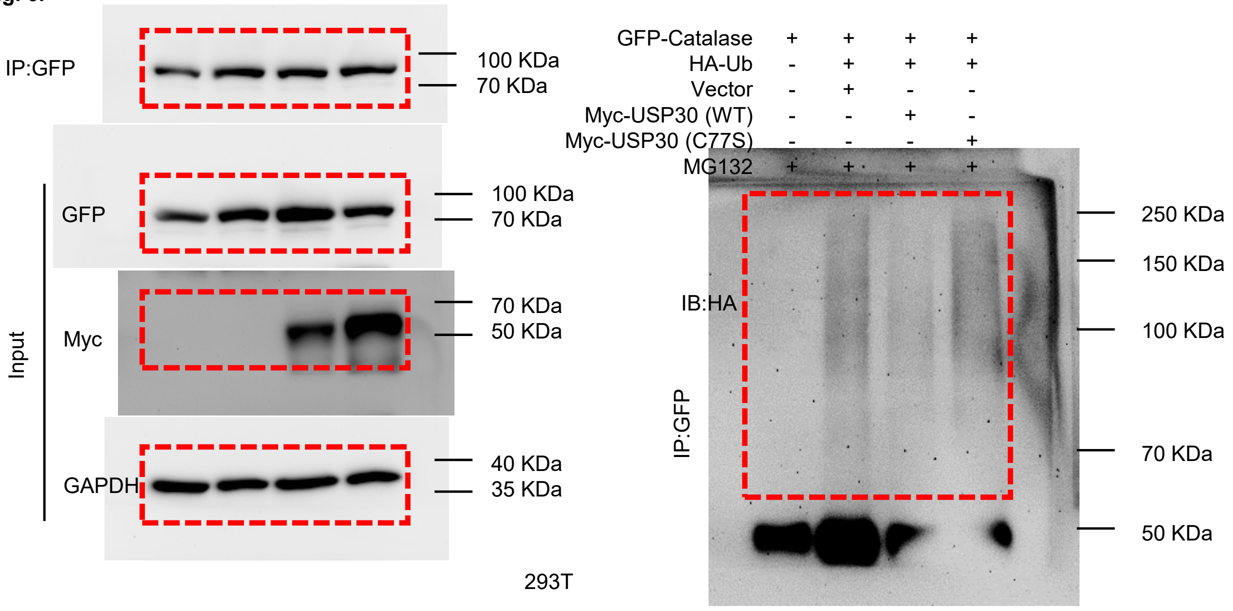

Supplementary Figure 10. Original uncropped Western blots.

Fig. 7j

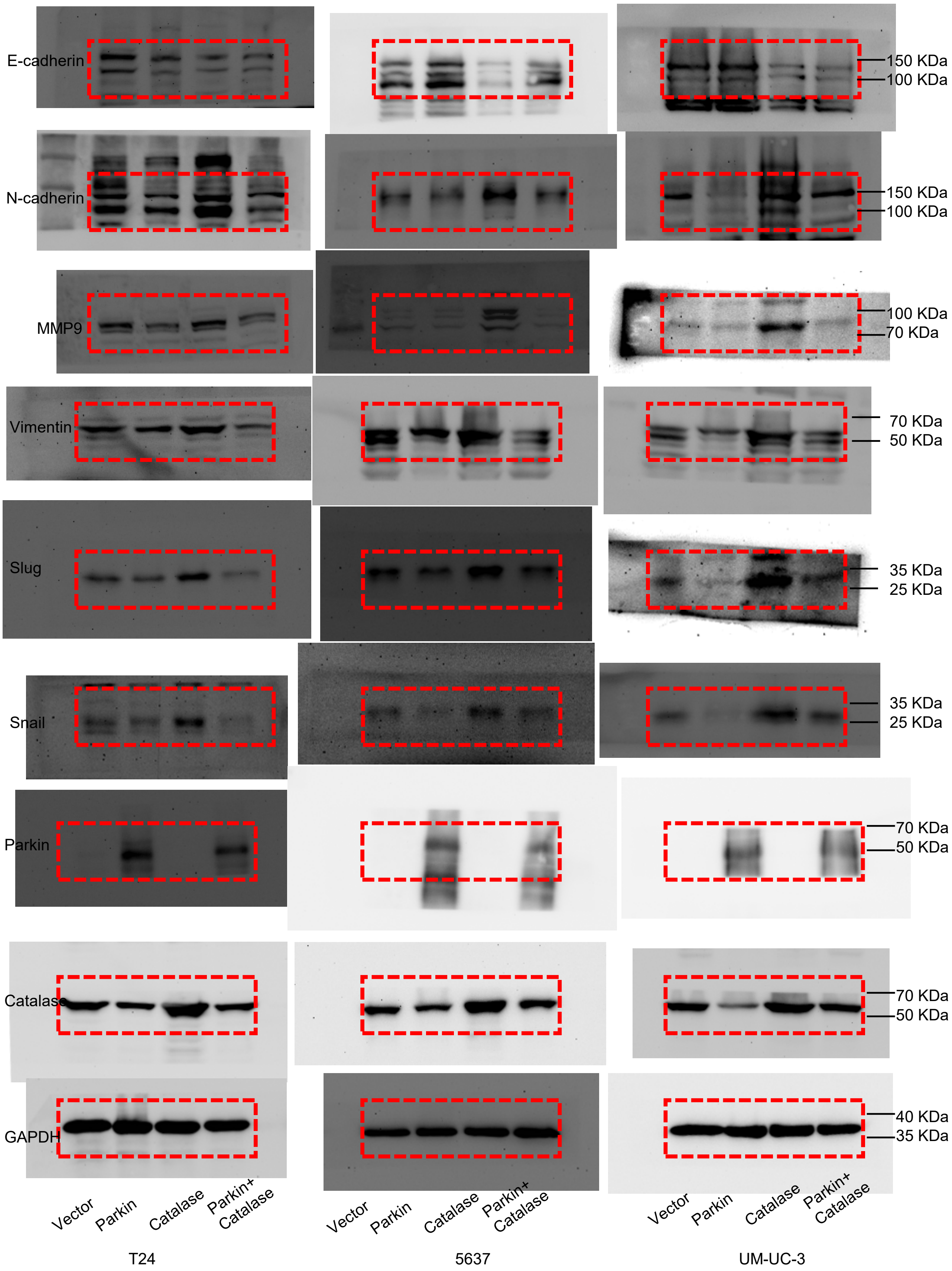

Supplementary Figure 10. Original uncropped Western blots.

Supplementary Fig. 1i

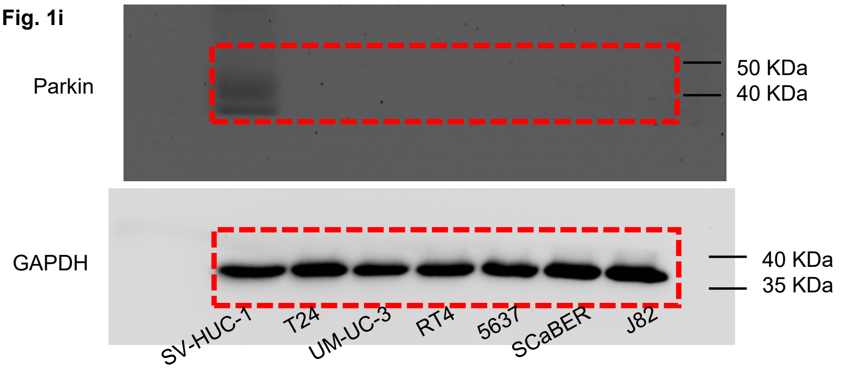

Supplementary Fig. 2a

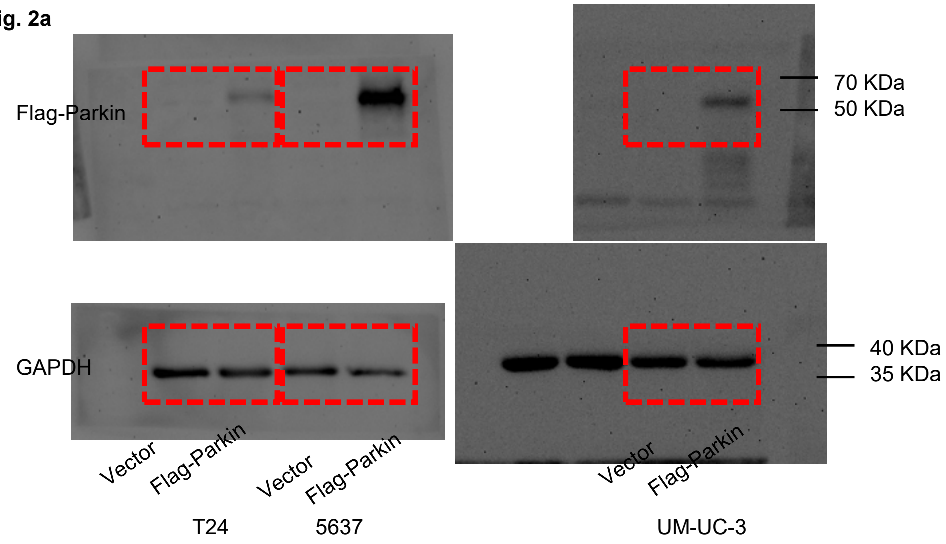

Supplementary Fig. 3d

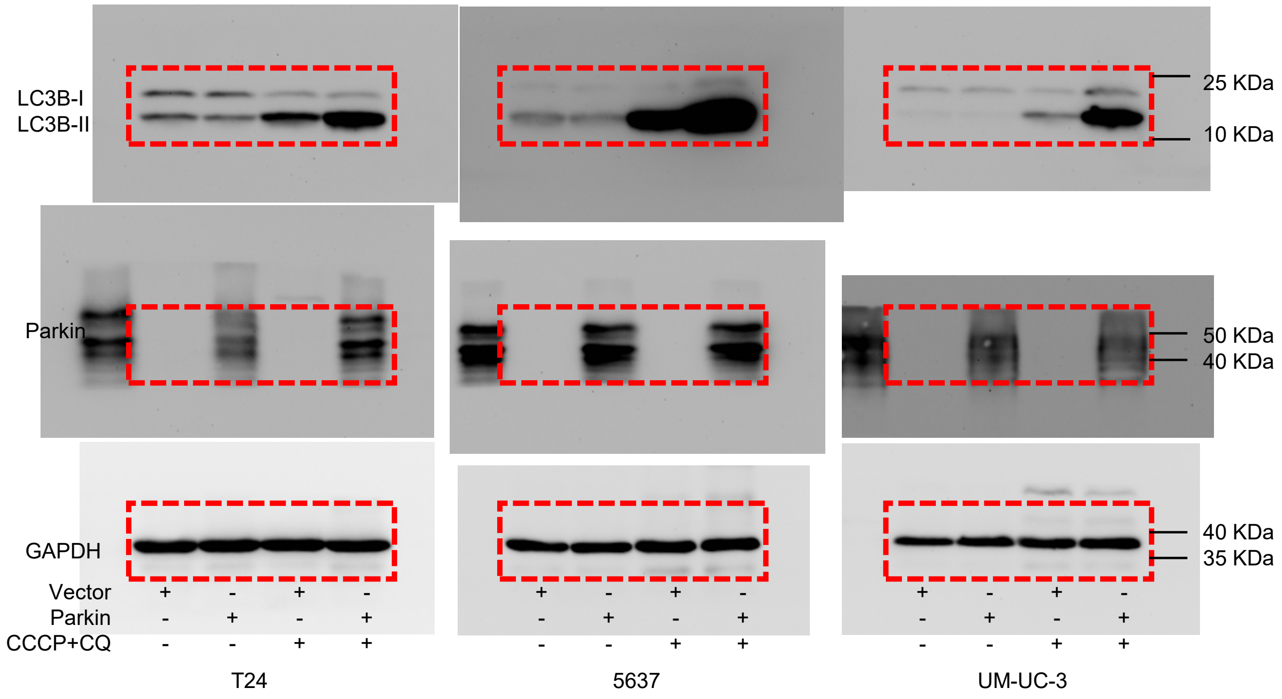

Supplementary Figure 10. Original uncropped Western blots.

Supplementary Fig. 5a

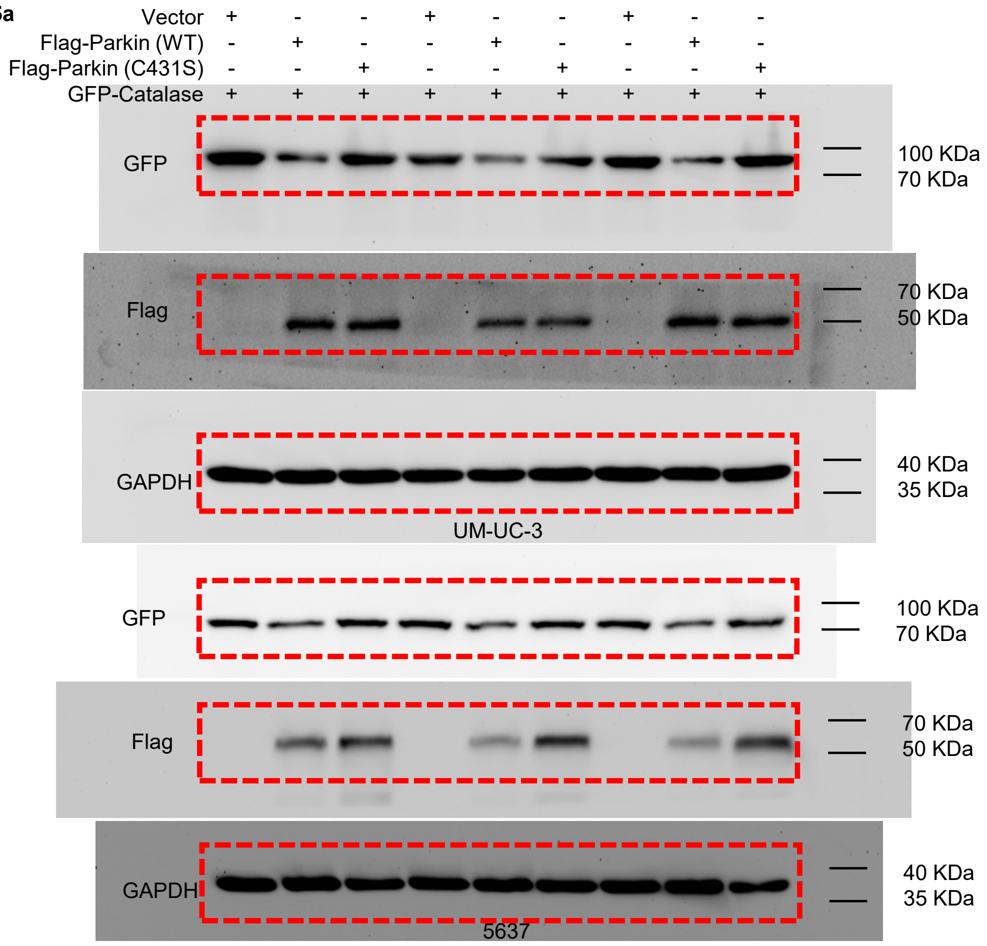

Supplementary Fig. 5c

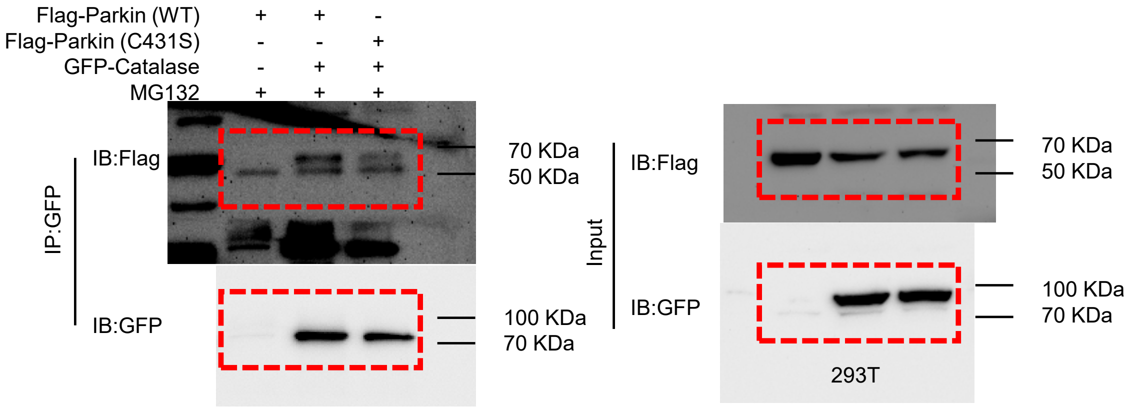

Supplementary Figure 10. Original uncropped Western blots.

Supplementary Fig. 5d

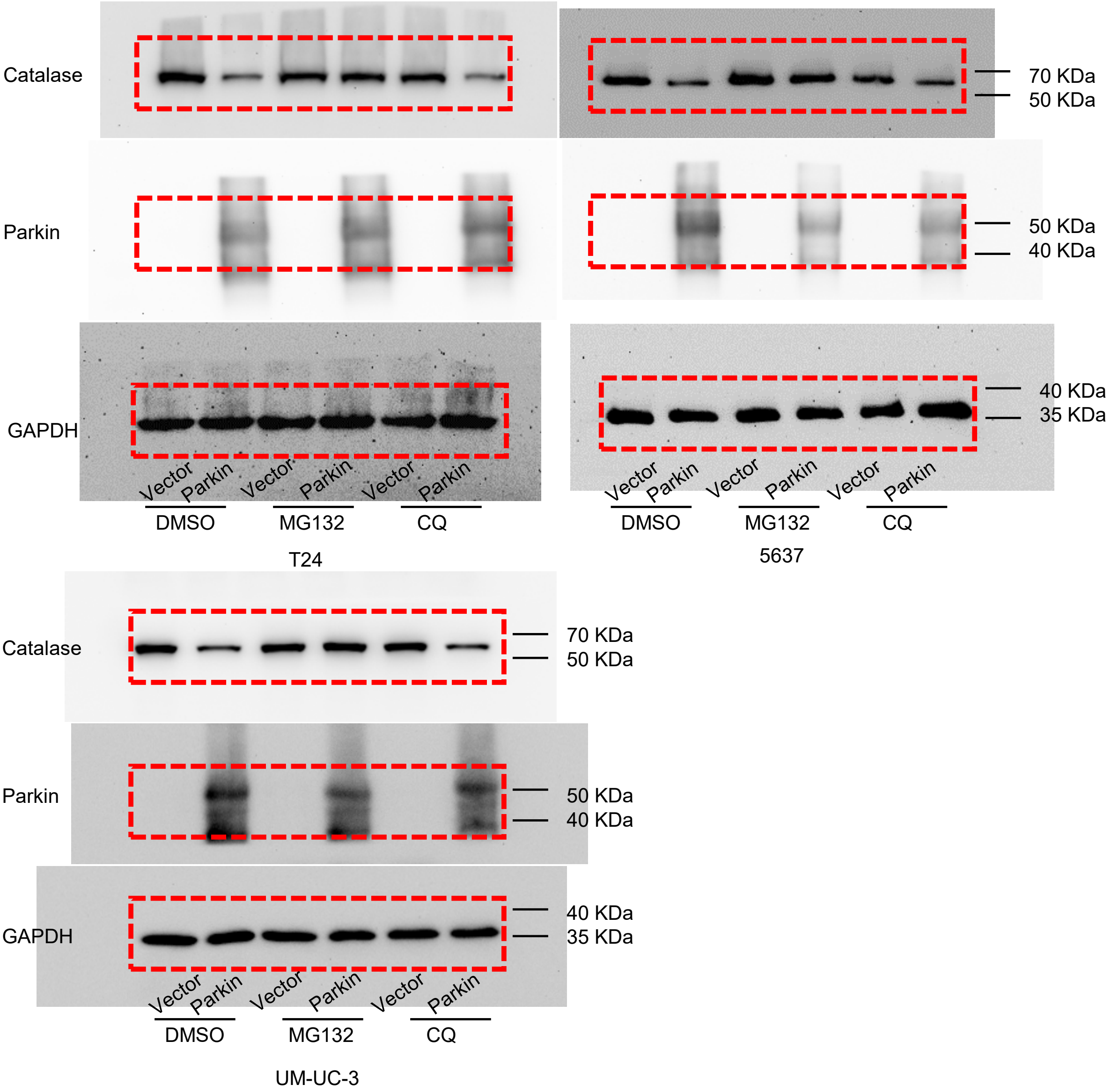

Supplementary Fig. 6a

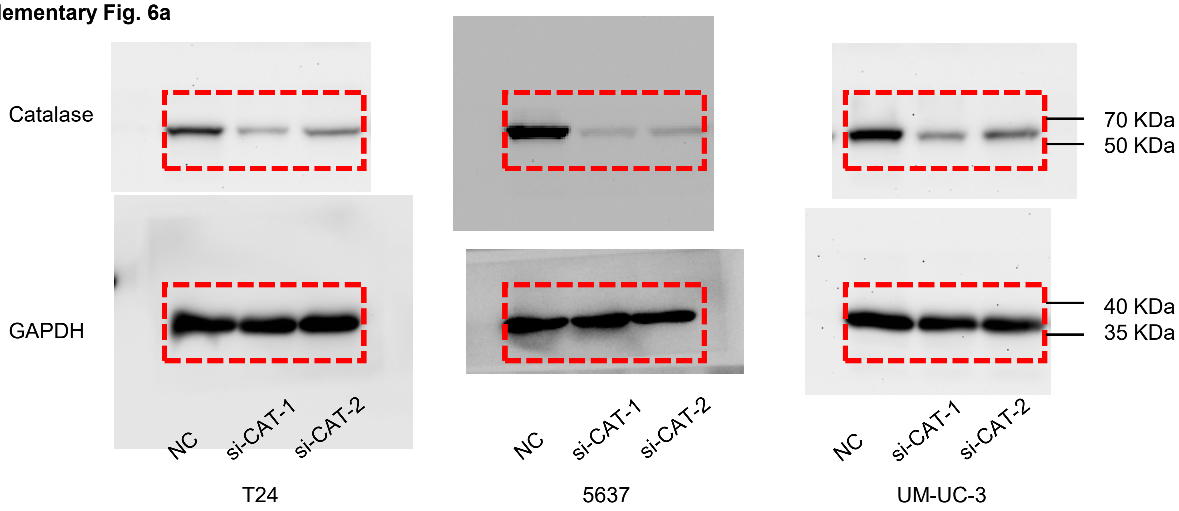

Supplementary Tables 1-4

Supplementary Table 1. Clinical characteristics of BLCA.

	***PRKN* expression**			
**Characteristics**	**Low**	**High**	**Chi-square**	***p* value**
Age				
≤ 65	10	10	0.432	0.511
> 65	16	23		
Gender				
Male	21	30	1.276	0.284
Female	5	3		
Pathological grade				
Low	0	6	5.262	0.03
High	26	27		
Muscle invasion				
No	2	10	5.134	0.023
Yes	24	21		
T stage				
Ta-T1	4	12	4.135	0.042
T2-T4	22	18		
N stage				
N0	21	24	0.23	0.715
N1-N3	3	5		

Note: Two cases were missing in "Muscle invasion"; Three cases were missing in "T stage"; Six cases were missing in "N stage".

Supplementary Table 2. Univariate Cox analysis.

	B	SE	Wald	df	Sig.	Exp(B)	95% CI	
							down	up
PRKN	-0.45	0.216	4.347	1	0.037	0.638	0.418	0.973
Gender	0.39	0.622	0.393	1	0.531	1.477	0.436	4.998
Age	-0.034	0.03	1.306	1	0.253	0.966	0.911	1.025
Pathological grade	-4.789	1.526	9.854	1	0.002	0.008	0	0.166
Muscle invasion	5.809	2.403	5.845	1	0.016	333.268	3.004	36978.509
T stage	0.171	0.859	0.04	1	0.842	1.187	0.221	6.385
N stage	1.365	0.638	4.575	1	0.032	3.916	1.121	13.678

Note: B: Beta coefficient, SE: Standard Error, Wald: Wald's chi-square value, df: degree of freedom, Sig.: significance, Exp(B): The exponent of B, CI: confidence interval.

Supplementary Table 3. Multivariate Cox analysis.

	B	SE	Wald	df	Sig.	Exp(B)	95% CI	
							down	up
PRKN	-0.48	0.209	5.267	1	0.022	0.619	0.411	0.932
Pathological grade	-4.259	1.442	8.721	1	0.003	0.014	0.001	0.239
Muscle invasion	5.444	1.814	9.007	1	0.003	231.409	6.612	8099.55
N stage	1.189	0.606	3.849	1	0.05	3.283	1.001	10.768

Note: B: Beta coefficient, SE: Standard Error, Wald: Wald’s chi-square value, df: degree of freedom, Sig.: significance, Exp(B): The exponent of B, CI: confidence interval.

Supplementary Table 4. Information for the primary antibodies used in this study.

Antibody	Supplier	Catalog	Application
Ki-67	Abcam	ab15580	1 μg/ml for IHC
Parkin	Santa Cruz	sc-32282	1:100 for IHC
Parkin	CST	4211S	1:1000 for WB; 1 μg/ml for IP
Catalase	Abcam	ab76024	1:2000 for WB; 1:100 for IHC
E-Cadherin	Proteintech	20874-1-AP	1:2000 for WB
N-Cadherin	Proteintech	22018-1-AP	1:2000 for WB
MMP-9	CST	13667S	1:1000 for WB
Vimentin	CST	5741S	1:1000 for WB
Slug	CST	9585S	1:1000 for WB
Snail	CST	3879S	1:1000 for WB
SQSTM1/p62	Abcam	ab56416	1:1000 for WB
LC3B	CST	2775S	1:1000 for WB
GAPDH	Proteintech	60004-1-Ig	1:10000 for WB
Flag-Tag	Sigma	F1804	1:1000 for WB; 1 μg/ml for IP; 1:200 for IF
GFP-Tag	ABclonal	AE012	1:1000 for WB
GFP-Tag	Santa Cruz	sc-9996	1 μg/ml for IP
Myc-Tag	ABclonal	AE010	1:1000 for WB; 1 μg/ml for IP
HA-Tag	Origene	TA180128	1:1000 for WB
