# Supplementary material for: Parkin inhibits proliferation and migration of bladder cancer via ubiquitinating Catalase
Source: Commun Biol. 2024 Feb 29;7:245. doi: 10.1038/s42003-024-05935-x (PMC10904755; doi:10.1038/s42003-024-05935-x)
Supplement: Supplementary file 4 — Reporting Summary [file 42003_2024_5935_MOESM4_ESM.pdf]

nature portfolio

Corresponding author(s): Tongzu Liu, Lingao Ju, Xinghuan Wang

Last updated by author(s): Jan 18, 2024

Reporting Summary

Nature Portfolio wishes to improve the reproducibility of the work that we publish. This form provides structure for consistency and transparency in reporting. For further information on Nature Portfolio policies, see our Editorial Policies and the Editorial Policy Checklist.

Statistics

For all statistical analyses, confirm that the following items are present in the figure legend, table legend, main text, or Methods section.

n/a	Confirmed	
☐	☒	The exact sample size (*n*) for each experimental group/condition, given as a discrete number and unit of measurement
☐	☒	A statement on whether measurements were taken from distinct samples or whether the same sample was measured repeatedly
☐	☒	The statistical test(s) used AND whether they are one- or two-sided<br>*Only common tests should be described solely by name; describe more complex techniques in the Methods section.*
☒	☐	A description of all covariates tested
☐	☒	A description of any assumptions or corrections, such as tests of normality and adjustment for multiple comparisons
☐	☒	A full description of the statistical parameters including central tendency (e.g. means) or other basic estimates (e.g. regression coefficient) AND variation (e.g. standard deviation) or associated estimates of uncertainty (e.g. confidence intervals)
☐	☒	For null hypothesis testing, the test statistic (e.g. *F*, *t*, *r*) with confidence intervals, effect sizes, degrees of freedom and *P* value noted<br>*Give P values as exact values whenever suitable.*
☒	☐	For Bayesian analysis, information on the choice of priors and Markov chain Monte Carlo settings
☒	☐	For hierarchical and complex designs, identification of the appropriate level for tests and full reporting of outcomes
☒	☐	Estimates of effect sizes (e.g. Cohen's *d*, Pearson's *r*), indicating how they were calculated

Our web collection on statistics for biologists contains articles on many of the points above.

Software and code

Policy information about availability of computer code

Data collection	The qRT-PCR data: StepOnePlus™ Real-Time PCR System (Thermo Fisher);<br>The WB imaging: chemiluminescence and gel imager (BioSpectrum 515 Imaging System, UVP);<br>The immunofluorescence images: the confocal laser microscope (C2, Nikon, Japan).<br>The animal fluorescence detection experiment: IVIS Lumina XRMS Series III (PerkinElmer).
Data analysis	All statistical analyses of the study were performed by GraphPad Prism software (version 7) software;<br>All WB and immunohistochemical (IHC) staining and immunofluorescence (IF) images quantitative analysis were performed by Image J software (version 1.51);<br>Gene Set Enrichment Analysis (GSEA) was performed by GSEA software (version 4.1.0).

For manuscripts utilizing custom algorithms or software that are central to the research but not yet described in published literature, software must be made available to editors and reviewers. We strongly encourage code deposition in a community repository (e.g. GitHub). See the Nature Portfolio guidelines for submitting code & software for further information.

Data

Policy information about availability of data

All manuscripts must include a data availability statement. This statement should provide the following information, where applicable:

- Accession codes, unique identifiers, or web links for publicly available datasets
- A description of any restrictions on data availability
- For clinical datasets or third party data, please ensure that the statement adheres to our policy

The publicly available TCGA-BLCA cohort data (the data included 408 tumors, and 19 normal samples) were obtained from the GDC Data Portal website (https://portal.gdc.cancer.gov/). The publicly available GSE data sets (GSE128959, GSE13507, GSE169455, GSE19423, GSE3167, GSE32548, GSE48075, GSE48276, GSE69795, GSE70691, GSE86411) were obtained from the National Center for Biotechnology Information website (https://www.ncbi.nlm.nih.gov/gds/). The remaining data are available within the Article, Supplementary Information or Source Data file. Source data are provided as a Source Data file.

Research involving human participants, their data, or biological material

Policy information about studies with human participants or human data. See also policy information about sex, gender (identity/presentation), and sexual orientation and race, ethnicity and racism.

Reporting on sex and gender	We randomly chose the tumor tissues based on the availability and not based on sex or gender.
Reporting on race, ethnicity, or other socially relevant groupings	NA
Population characteristics	We randomly chose the tumor tissues based on the availability and not based on Population characteristics.
Recruitment	We recruited individuals diagnosed with bladder cancer (BLCA) and scheduled for surgical resection and from whom we obtained informed consent. Given the small number of patients our cohort might not encompass the molecular diversity of human bladder cancer and the results might not apply to every bladder tumor.
Ethics oversight	The study was approved by the Institutional Ethics Committee of Zhongnan Hospital of Wuhan University.

Note that full information on the approval of the study protocol must also be provided in the manuscript.

Field-specific reporting

Please select the one below that is the best fit for your research. If you are not sure, read the appropriate sections before making your selection.

☒ Life sciences ☐ Behavioural & social sciences ☐ Ecological, evolutionary & environmental sciences

For a reference copy of the document with all sections, see nature.com/documents/nr-reporting-summary-flat.pdf

Life sciences study design

All studies must disclose on these points even when the disclosure is negative.

Sample size	Samples size for each experiment is indicated in the figures or corresponding figure legends.
Data exclusions	No samples or animals were excluded from the analyses.
Replication	The experiments were successfully repeated. Clear statements have been put into Methods section and Figure legends.
Randomization	The mice were randomly put into separate/groups cages for experiments.
Blinding	For mice studies, the experiments were performed in a blinded fashion when possible. Downstream analyses of mice samples (IHC and H&E) were performed in a blinded fashion, which means that people performing the assays were not aware of the treatment groups until the data analyses were completed.

Reporting for specific materials, systems and methods

We require information from authors about some types of materials, experimental systems and methods used in many studies. Here, indicate whether each material, system or method listed is relevant to your study. If you are not sure if a list item applies to your research, read the appropriate section before selecting a response.

Materials & experimental systems

n/a	Involved in the study	
☐	☒	Antibodies
☐	☒	Eukaryotic cell lines
☒	☐	Palaeontology and archaeology
☐	☒	Animals and other organisms
☒	☐	Clinical data
☒	☐	Dual use research of concern
☒	☐	Plants

Methods

n/a	Involved in the study	
☒	☐	ChIP-seq
☐	☒	Flow cytometry
☒	☐	MRI-based neuroimaging

Antibodies

Antibodies used

Target, Catalog No., Supplier, Application/Dilution or amount

For Western blot experiment:
Anti-Parkin, 4211S ,Cell Signaling Technology, WB/1:1000,IP/1 μg
Anti-Catalase, ab76024, Abcam, WB/1:1500-3000
Anti-E-Cadherin, 20874-1-AP, Proteintech, WB/1:5000
Anti-N-Cadherin, 22018-1-AP, Proteintech, WB/1:2000
Anti-MMP-9, 13667S, Cell Signaling Technology, WB/1:1000
Anti-Vimentin, 5741S, Cell Signaling Technology, WB/1:1000
Anti-Slug, 9585S, Cell Signaling Technology, WB/1:1000
Anti-Snail, 3879S, Cell Signaling Technology, WB/1:1000
Anti-SQSTM1/p62, ab56416, Abcam, WB/1:1000
Anti-LC3B, 2775S, Cell Signaling Technology, WB/1:1000
Anti-GAPDH, 60004-1-Ig, Proteintech, WB/1:5000
Anti-Flag, F1804, Sigma, WB/1:1000,IP/1 μg
Anti-GFP-Tag, AE012, ABclonal, WB/1:1000
Anti-Myc-Tag, AE010, ABclonal, WB/1:1000,IP/1 μg
Anti-GFP-Tag, sc-9996, Santa Cruz, IP/1 μg

For immunohistochemistry staining:
Anti-Ki-67, ab15580, Abcam, 1:100
Anti-Parkin(PRK8), sc-32282, Santa Cruz, 1:100
Anti-Catalase, ab76024, Abcam, 1:100

For immunofluorescence staining:
Anti-Flag, F1804, Sigma, IF/1:200
405-conjugated Goat Anti-Mouse IgG(H+L), ABclonal, IF/1:1000

Validation

All antibodies were purchased from commercial companies, and validated by the data sheets of themanufacturer or citations listed below.
The following primary antibodies were used for Western blot experiments:
1) Anti-Parkin, validated with Western blot analysis of extracts from PC12 cells, fetal rat brain and mouse brain (https://www.cellsignal.com/products/primary-antibodies/parkin-prk8-mouse-mab/4211);
2) Anti-Catalase, validated with Western blot analysis of extracts from HeLa cell lysate (https://www.abcam.cn/products/primary-antibodies/catalase-antibody-ep1929y-peroxisome-marker-ab76024.html);
3) Anti-E-Cadherin, validated with Western blot analysis of extracts from A431 cells, DU 145 cells, mouse testis tissue, HCT 116 cells, MCF-7 cells, T-47D cells (https://www.ptgcn.com/Products/E-cadherin-Antibody-20874-1-AP.htm);
4) Anti-N-Cadherin, validated with Western blot analysis of extracts from mouse brain tissue, rat brain tissue, HEK-293 cells, C2C12 cells, C6 cells, rat heart tissue, HeLa cells, PC-3 cells(https://www.ptgcn.com/products/N-cadherin-Antibody-22018-1-AP.htm);
5) Anti-MMP-9, validated with Western blot analysis of extracts from U-2 OS cells (https://www.cellsignal.com/products/primary-antibodies/mmp-9-d6o3h-xp-rabbit-mab/13667?site-search-type=Products&N=4294956287&Ntt=+13667s&fromPage=plp&_requestid=3607864);
6) Anti-Vimentin, validated with Western blot analysis of extracts from MCF-7, Hela, RD and COS7 whole cell lysates (https://www.cellsignal.cn/products/primary-antibodies/ldha-c4b5-rabbit-mab/3582?site-search-type=Products&N=4294956287&Ntt=3582&fromPage=plp&_requestid=2199715);
7) Anti-N-cadherin, validated with Western blot analysis of extracts from A549, PC-3, HCT 116 and HepG2 whole cell lysates (https://www.abcam.cn/products/primary-antibodies/n-cadherin-antibody-epr1791-4-ab76011.html#lb);
8) Anti-Vimentin, validated with Western blot analysis of extracts from Hela, NIH/3T3, C6 and COS-7 whole cell lysates (https://www.cellsignal.com/products/primary-antibodies/vimentin-d21h3-xp-rabbit-mab/5741?site-search-type=Products&N=4294956287&Ntt=5741s&fromPage=plp&_requestid=3608511);
9) Anti-Slug, validated with Western blot analysis of extracts from A204, SKMEL5 and NIH/3T3 whole cell lysates (https://www.cellsignal.cn/products/primary-antibodies/slug-c19g7-rabbit-mab/9585?site-search-type=Products&N=4294956287&Ntt=9585s&fromPage=plp&_requestid=2120390);
10) Anti-Snail, validated with Western blot analysis of extracts from HCT116, Hela, NIH/3T3, Rat2 and COS7 whole cell lysates (https://www.cellsignal.cn/products/primary-antibodies/snail-c15d3-rabbit-mab/3879?site-search-type=Products&N=4294956287&Ntt=3879s&fromPage=plp&_requestid=2201814);
11) Anti-SQSTM1/p62, validated with Western blot analysis of extracts from Human Tissue lysate, Mouse Cell lysate, neuroblastoma,

DU145 Prostate cancer cell line (https://www.abcam.cn/products/primary-antibodies/sqstm1--p62-antibody-2c11-bsa-and-azide-free-ab56416.html);
12) Anti-LC3B, validated with Western blot analysis of extracts from HeLa cells, HT-1080 and A20 cells (htt12) Anti-LC3B, validated with Western blot analysis of extracts from HeLa cells, HT-1080 and A20 cells (https://www.cellsignal.com/products/primary-antibodies/lc3b-antibody/2775);ps://www.cellsignal.com/products/primary-antibodies/lc3b-antibody/2775);
13) Anti-GAPDH, validated with Western blot analysis of extracts from HeLa cells, HepG2 cells, ROS1728 cells, pig brain tissue, zebrafish tissue, whole yeast, whole Nematode tissue, soybean whole plant tissue, arabidopsis whole plant tissue, HEK-293 cells, Jurkat cells, K-562 cells, HSC-T6 cells, NIH/3T3 cells, 4T1 cells, C6 cells, PC-12 cells, C2C12 cells, SP2/0 cells, rat brain tissue, mouse brain tissue (https://www.ptgcn.com/products/GAPDH-Antibody-60004-1-Ig.htm);
14) Anti-Flag-Tag, validated with Western blot analysis of Flag protein in CHO lysis solution (https://www.sigmaaldrich.cn/deepweb/assets/sigmaaldrich/product/documents/144/194/vol6_iss2_antiflag_m2.pdf);
15) Anti-GFP-Tag, validated with Western blot analysis of insect expressed GFP protein(https://abclonal.com.cn/catalog/AE012);
16)Anti-Myc-Tag, validated with Western blot analysis of extracts from 293T transfected with Myc-NLK protein (https://abclonal.com.cn/catalog/AE010);

The following primary antibodies were used for immunohistochemistry staining:
1) Anti-Ki-67, validated with immunohistochemical analysis (Formalin/PFA-fixed paraffin-embedded sections) of Mouse Tissue sections (tumor) Human Tissue sections (U87MG cell) (https://www.abcam.cn/products/primary-antibodies/ki67-antibody-ab15580.html);
2) Anti-Parkin, validated with immunohistochemical analysis of formalin fixed, paraffin-embedded human gall bladder tissue showing cytoplasmic and nuclear staining of glandular cells (https://www.scbt.com/zh/p/parkin-antibody-prk8?requestFrom=search);
3) Anti-Catalase, validated with immunohistochemical analysis of Paraffin-embedded human bladder cancer tissue sections (https://www.abcam.cn/products/primary-antibodies/catalase-antibody-ep1929y-peroxisome-marker-ab76024.html);

For immunofluorescence staining:
1) Anti-Flag-Tag, validated with immunofluorescence analysis of Flag protein in CHO lysis solution (https://www.sigmaaldrich.cn/deepweb/assets/sigmaaldrich/product/documents/144/194/vol6_iss2_antiflag_m2.pdf);
2) 405-conjugated Goat Anti-Mouse IgG(H+L), secondary antibodies are generated by immunizing the host animal (different from host species of primary antibody) with a pooled population of normal immunoglobulins from the host species of primary antibody and can be further purified and modified (i.e. antibody fragmentation, label conjugation, etc.) to ensure well-characterized specificity to corresponding normal immunoglobulins (https://abclonal.com.cn/catalog/AS055)

Eukaryotic cell lines

Policy information about cell lines and Sex and Gender in Research

Cell line source(s)	T24, 5637, UM-UC-3, and HEK 293T cells were kindly provided by Cell Bank of Chinese Academy of Science (Shanghai, China).
Authentication	Authentication was performed by Cell Bank, Chinese Academy of Sciences (Shanghai, China).
Mycoplasma contamination	All cell lines were tested negative for mycoplasma contamination.
Commonly misidentified lines (See ICLAC register)	None.

Animals and other research organisms

Policy information about studies involving animals; ARRIVE guidelines recommended for reporting animal research, and Sex and Gender in Research

Laboratory animals	We purchased 36 male BALB/c nude mice (6 weeks old) from WQJX Bio Technology (Wuhan, China) and adaptively fed them in a specific pathogen-free (SPF) facility for 1 week.
Wild animals	No wild animals were used in this study.
Reporting on sex	The mice used in the study were all male, because the incidence of bladder cancer is much higher in men than in women (PMID: 27370177).
Field-collected samples	No field-collected samples were used in this study.
Ethics oversight	All work with mice was approved by and performed under the regulations of the Experimental Animal Welfare and Ethics Committee at Zhongnan Hospital of Wuhan University (ZN2022242).

Note that full information on the approval of the study protocol must also be provided in the manuscript.

Plants

Seed stocks	NA.
Novel plant genotypes	NA.
Authentication	NA.

Flow Cytometry

Plots

Confirm that:

☒ The axis labels state the marker and fluorochrome used (e.g. CD4-FITC).

☒ The axis scales are clearly visible. Include numbers along axes only for bottom left plot of group (a 'group' is an analysis of identical markers).

☒ All plots are contour plots with outliers or pseudocolor plots.

☒ A numerical value for number of cells or percentage (with statistics) is provided.

Methodology

Sample preparation	Transfected BLCA cells were harvested and washed twice with cold PBS, followed by centrifugation. The cells were then suspended using DCFH-DA (10 μM) probe, JC-1 detection kit or the annexin V-FITC apoptosis kit (Sungene), respectively. and incubated in the dark for 30 min at room temperature. Finally, Flow cytometry (Beckman Cytoflex) was used to detect the samples. CytExpert 2.4 and FlowJo v10.8.1 were used to analyze the results.
Instrument	Flow cytometry (Beckman Cytoflex)
Software	CytExpert 2.4 and FlowJo v10.8.1 were used to analyze the results.
Cell population abundance	No sorting was performed.
Gating strategy	Intact cells were gated in the forward scatter/side scatter (FSC/SSC) plot to exclude small fragments.

☒ Tick this box to confirm that a figure exemplifying the gating strategy is provided in the Supplementary Information.
